# Nanobodies against *Clostridioides difficile* CDTb provide a toolkit for potent toxin neutralization and highly sensitive quantitation

**DOI:** 10.1016/j.jbc.2025.111082

**Published:** 2025-12-22

**Authors:** Kateryna Nabukhotna, Heather K. Kroh, David M. Anderson, Rubén Cano Rodríguez, John A. Shupe, Maria McGresham, Carla V.T. O’Neale, Rebecca A. Shrem, Brian E. Wadzinski, Kevin L. Schey, Benjamin W. Spiller, D. Borden Lacy

**Affiliations:** 1 Department of Pathology, Microbiology, and Immunology, Vanderbilt University Medical Center, Nashville, Tennessee, USA; 2 Department of Pharmacology, Vanderbilt University, Nashville, Tennessee, USA; 3 Carterra, Salt Lake City, Utah, USA; 4 Department of Biochemistry, Vanderbilt University, Nashville, Tennessee, USA; 5 Department of Veterans Affairs, Tennessee Valley Healthcare System, Nashville, Tennessee, USA

**Keywords:** toxin, nanobody, epitope characterization, *C. difficile*, protein–protein interactions, molecular mechanism

## Abstract

*Clostridioides difficile* is a pathogenic bacterium and a leading cause of antibiotic-associated diarrhea. Symptoms of the infection arise because of the production of large clostridial toxins that disrupt the intestinal barrier and cause an acute host inflammatory response. Epidemic *C. difficile* strains also produce the *C. difficile* transferase toxin (CDT), a binary toxin consisting of separate enzymatically active (CDTa) and cell-binding (CDTb) components. However, the role of CDT during *C. difficile* pathogenesis remains poorly understood. We created a CDTb nanobody (Nb) clone library and identified and purified five clones with promising CDTb-binding properties. Studies using the Carterra LSA^XT^ platform revealed high-affinity binding interactions between the Nbs and three distinct CDTb epitopes. Functionally, these Nbs potently neutralize cellular cytopathic effects of CDT at equimolar concentrations *in vitro*. We further identified two distinct neutralization mechanisms—inhibition of CDTb heptamer formation and inhibition of cell surface binding, both of which are crucial for CDTa delivery into the host cell. These Nbs were used in a sandwich ELISA assay to monitor CDTb levels between 1- and 7-day post R20291 infection in the cecal material of infected mice. Notably, levels of CDTb spiked during days 3 and 4, with monomers constituting the majority of CDTb. We anticipate that these reagents will allow researchers to further expand toxin intervention and monitoring strategies to obtain a deeper understanding of the CDT mechanism of action.

*Clostridioides difficile* is a leading cause of nosocomial diarrhea in the United States and is classified as a “threat-level urgent” pathogen by the US Centers for Disease Control (1, 2, 3). Most frequently, the disease occurs because of disruption of the host microbiome in response to antibiotic treatment and is characterized by mild to severe (and often recurrent) diarrhea. The progression of *C. difficile* infection (CDI) can lead to pseudomembranous colitis, toxic megacolon, sepsis, and even death (4).

In addition to risk factors, such as a patient’s age, antibiotic exposure, and underlying health and immune conditions, symptomatic CDI is dependent on the secretion of up to three toxins: toxin A (TcdA), toxin B (TcdB), and the *C. difficile* transferase toxin (CDT, or binary toxin) (1). While TcdA and TcdB are the primary virulence factors (5), epidemic *C. difficile* strains (*e.g.*, NAP1/BI/027) also produce CDT, suggesting that it may be important for the severity of CDI (6, 7, 8). Indeed, several studies indicate that the presence of the *cdtB* gene is associated with worse infection outcomes, such as higher fatality rates and an increased rate of recurrence (9, 10). In addition, inclusion of binary toxin during vaccine development significantly increased survival in hamsters compared with a vaccine containing only TcdA and TcdB antigens (11, 12). The prevalence of clinical isolates harboring CDT and the potential for developing the toxin as an effective vaccine antigen necessitate a deeper understanding of neutralizing toxin epitopes.

The CDT structure and cellular mechanism of action have been historically compared with *Clostridium perfringens* iota toxin, *Bacillus anthracis* anthrax toxin, and other clostridial binary toxins because of their structural homology (13). Within the last decade, several studies have offered new insights into CDT biology, and structures of CDTb and CDTa alone and in complex have now been extensively characterized by us and others (14, 15, 16, 17, 18, 19). CDT is a binary toxin consisting of two proteins: an ADP-ribosyltransferase (CDTa) and a cell-binding and pore-forming protein (CDTb) that translocates CDTa into the host cytosol (20). CDTb consists of five domains (D1–D3, D3′, and D4) and one linker (14). D1 (residues 43–295) holds CDTb in a monomeric form until it undergoes proteolytic activation. D2 (296–511) contains a pore-forming loop that can transform into a β-barrel pore and, together with D3 (512–615), makes up an oligomerization interface that allows CDTb to form a heptamer. D3′ (616–744) is a discrete domain composed of two β-sheets arranged in a β-roll fold that may bind to glycans based on the sequence homology studies (14). D4 (760–876) is a cell surface receptor–binding domain connected to the rest of the protein *via* a flexible 14-residue linker (745–759) (Fig. 1*A*). Once secreted into the gut lumen, CDTb engages host cells by binding the lipolysis-stimulated lipoprotein receptor. Full-length CDTb monomer (98 kDa) is cleaved by serine proteases on the cell surface, resulting in the release of the ∼20 kDa D1′ domain. The remaining ∼75 kDa-activated CDTb monomer can then oligomerize into a heptameric prepore that can bind one CDTa molecule in the center of the heptamer (15). The CDTa–(CDTb)_7_ complex then gets endocytosed by the target cell, CDTb transitions from the prepore to the β-barrel lipid-inserted pore (Fig. 1*A*), and endosomal acidification leads to translocation of CDTa through the CDTb β-barrel pore with assistance of cytosolic chaperones (15, 21, 22). Once in the cytosol, CDTa promotes cytopathic changes through the transfer of ADP-ribose from NAD^+^ to Arg177 of monomeric G-actin (20). Depolymerization of the actin cytoskeletal structure has been shown to cause the formation of microtubule protrusions that increase *C. difficile* adherence on the surface of epithelial cells, explaining how CDT may increase the virulence of *C. difficile* (23, 24). It was recently proposed that CDT also induces the formation of biofilm-like mucin-associated microcolonies implicated in *C. difficile* colonization, persistence, and antibiotic resistance (25). These downstream consequences highlight the need for therapeutic strategies that can be used for neutralizing CDT function.

**Figure 1:**
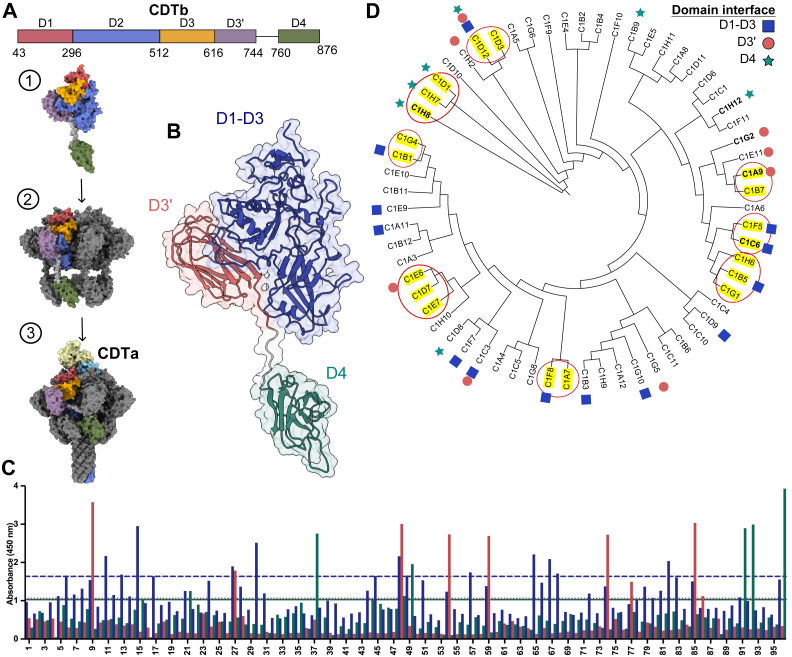
**The initial characterization of 94 lysates provided evidence of diverse sequences capable of recognizing three or more distinct epitopes on CDTb.***A,* color-coded schematic of CDTb domain organization and transition from the monomer (1) to prepore (2) to CDTa-bound pore (3) (PDB IDs: 6O2N and 6V1S). Pseudo-ADP ribosyltransferase (Padprt) and ADP ribosyltransferase (ADPRT) of CDTa are shown in *light blue* and *yellow*, respectively (PDB ID: 2WN6). *B,* after derivation of nanobody (Nb) clones, CDTb was expressed as three distinct truncation proteins for screening: D1–D3 (*blue*, residues 43–615), D3’ (*red*, residues 616–750), and D4 (*green*, residues 760–876). *C,* protein lysates of the 94-clone panel were tested for binding to CDTb_D1–D3_ (*blue bars*), CDTb_D3’_ (*red bars*), and CDTb_D4_ (*green bars*) by ELISA. The threshold level for each protein construct was defined as the average value of the negative controls (clones 1 and 2) multiplied by two and shown as horizontal lines in respective colors on the *Y*-axis. Clones with values above the thresholds were colored on the cladogram. The experiment was independently performed one time with one technical replicate. *D,* a cladogram representing the amino acid sequence alignments of the 63 Nb sequences obtained. Clones with identical sequences (eight groups) are highlighted in *yellow* and circled in *red*. The five Nbs that were further characterized in this study are shown in *bold font*. “C1” in front of each Nb correlates to the internal plate numbering, and for conciseness purposes, the “C1” plate identifier will be omitted throughout the rest of the article. Domain specificity was assigned based on ELISA results shown in *C* (D1–D3, *blue*; D3′, *red*; and D4, *green*). CDTa, *Clostridioides difficile* transferase toxin, A subunit; CDTb, *Clostridioides difficile* transferase toxin, B subunit; PDB, Protein Data Bank.

In addition to the value of identifying neutralizing epitopes and antitoxin reagents, there is a lack of quantitative diagnostic research tools to assess the levels of CDT in physiological samples. Most of the clinical studies only consider the presence of binary toxin genes within the CDT locus when exploring the impact of CDT on infection outcomes (6). However, it was previously shown that the presence of the *cdtB* gene measured by PCR is not indicative of the presence of CDTb protein in patient stool samples measured by ELISA (26). Specifically, only 19 of 36 (53%) *cdt*^*+*^ gene isolates had positive hits for CDTb protein in fecal specimens. In addition, isolates from fecal samples generally produced lower levels of CDTb when grown in broth culture compared with their levels in the fecal milieu. This is consistent with the idea that the regulation of toxin gene expression is highly dependent on environmental cues that vary *in vitro*, *in vivo*, and likely even along the gastrointestinal tract of humans (27). Thus, our understanding of toxin molecular mechanisms in animal models or human clinical infection hinges on quantitative measurements of toxin proteins in physiological samples. Notably, there are no commercial ELISAs available for CDTb and CDTa quantification. To our knowledge, only two CDTb-specific ELISAs have been reported for research purposes. The first, mentioned above, is based on murine monoclonal antibodies to a *C. perfringens* pore-forming component as a capture and goat anti-CDTb antibodies obtained *via* proprietary methods as a detection (26). The second ELISA measures CDTb concentrations by using commercial chicken antibodies (25).

Nanobodies (Nbs) derived from camelid heavy-chain–only antibodies have become an advantageous tool in biomedical research (28). The antigen-binding paratope of Nbs is composed of just one variable heavy chain domain (VHH), often containing a long complementarity-determining region 3 (CDR3), responsible for antigen recognition and specificity (29, 30). In addition, Nbs are small, stable, soluble, and can be recombinantly produced in large quantities. They can also be converted to multivalent and/or multispecific formats, and into full heavy-chain antibodies with Fc-mediated effector functions. Our group has recently created panels of Nbs that enabled the identification of neutralizing epitopes of TcdA and TcdB and toxin quantification in feces and cecal contents of infected mice using a sandwich ELISA format (31). Here, we present five CDTb-specific Nb clones, which can be used for similar applications.

## Results

### Creation of an anti-CDTb Nb panel

Peripheral blood mononuclear cells (PBMCs) were obtained from an alpaca immunized with purified CDTb (residues 43–876). VHH-phage display libraries were generated by reverse transcriptase and PCR amplification of the VHH repertoire from blood lymphocytes and cloned into a pADL22 vector. Phage-displaying CDTb-specific Nbs were selected by panning the phage library against immobilized CDTb. *Escherichia coli* were infected with the eluted phage and plated into a 96-well plate with the goal of isolating single clones. Lysates from the 94 experimental wells were used for protein ELISA screening and DNA sequencing. Specifically, the 94 protein lysates were tested for their binding properties to three distinct CDTb domain variants: CDTb_D1–D3_, CDTb_D3′_, and CDTb_D4_ (Fig. 1*B*). The ELISA-based screen revealed lysates with a strong signal against one or more of the proteins. Specifically, there were 13, 8, and 7 lysates with a strong signal against the D1–D3, D3′, and D4 constructs, respectively (Fig. 1*C*). In some cases, a lysate had a strong signal against multiple constructs, an indication of nonspecific binding, and/or the presence of multiple clones within the well. DNA sequencing did reveal evidence of some lysates containing multiple clones, and we did not go back to separate these into individual clones. Instead, we moved forward with the 63 DNA sequences that emerged from the analysis and performed alignments on their encoded amino acid sequences (Figs. 1*D*, S1, and Table S1). Their residue alignment, displayed as a cladogram in Figure 1*D*, revealed 52 unique sequences with 44 appearing once and 8 appearing two or three times (highlighted in yellow and circled in red).

We selected five Nb clones for further study. C6 was chosen because it appeared in a cluster of five highly related sequences and was predicted to bind the D1–D3 domains. A9 and G1 were chosen based on their strong positive ELISA signal against the D3′ domain and turned out to be closely related at the sequence level. H8 and H12 appeared to bind domain 4 but were located in distinct areas of the cladogram. The sequences were cloned into a mammalian expression vector with an AviTag at the C terminus to allow for the generation of biotinylated forms of the Nbs (Table S2). The five Nbs were expressed and purified to homogeneity.

### Binding kinetics between CDTb constructs and Nbs reveal tight protein interactions

**Figure 2:**
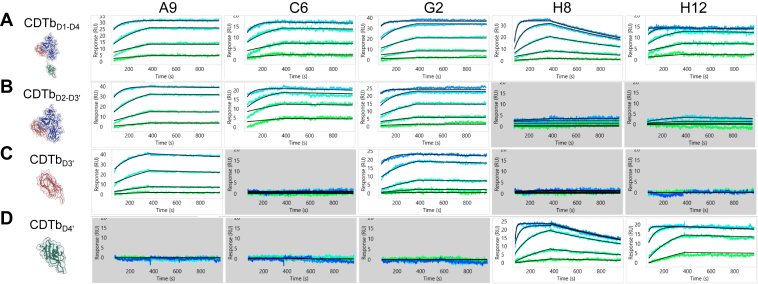
**Binding kinetics between CDTb constructs and nanobodies (Nbs) reveal tight protein interactions.** Representative surface plasmon resonance (SPR) sensorgrams of (*A*) CDTb_D1–D4_, (*B*) CDTb_D2–D3’_, (*C*) CDTb_D3’_, and (***D***) CDTb_D4_ injected over immobilized A9, C6, G2, H8, and H12 Nbs. Data shown come from different ligand (Nb) densities: higher ligand density regions of interest (ROIs) are shown for low molecular weight (MW) constructs like CDTb_D3’_ and CDTb_D4_, whereas low ligand density ROIs are shown for CDTb_D1–D4_ and CDTb_D2–D3’_. Analyte injections are shown in a *green–blue palette*, with the lowest analyte concentration in *green* and the highest in *blue*. Fits to one-to-one kinetics model are shown in *black*. Noninteracting pairs are highlighted in *gray*. Summary of interactions is shown in Table 1, Table 2, Table 3, Table 4. The experiment was independently performed one time with three to four technical replicates. CDTb, *Clostridioides difficile* transferase toxin, B subunit; ROI, region of interest.

We then tested the purified Nbs for their capacity to bind CDTb. The high-throughput surface plasmon resonance (SPR) Carterra LSA^XT^ platform was used to determine binding kinetics and affinity between our lead Nbs (ligands) and CDTb (analyte). The Carterra LSA^XT^ system enables high-throughput, one-on-many assay formats using a combination of multichannel and single-channel microfluidics. A 96-channel printhead can immobilize 96 ligands at a time and perform up to four nested prints, enabling preparation of arrays containing up to 384 unique ligands. The single-channel flow cell then delivers analyte solutions across the 384-ligand array along with 48 reference spots. Binding events are monitored in real time by a CCD camera that measures resonance shifts at each ligand location simultaneously, allowing detection of up to 384 interactions at once. The high-throughput features of the platform allowed us to immobilize the Nb ligands in quadruplicate at eight different concentrations and allowed us to test multiple CDTb constructs. In addition to using the complete CDTb_D1–D4_ monomer (residues 43–876) as an analyte, we also included CDTb_D2–D3′_ (210–749), CDTb_D3′_ (615–760), and CDTb_D4_ (760–876) truncations to confirm domain epitope specificity of the Nbs. SPR analysis revealed tight binding affinities (18–50 pM) between four of the Nbs and full-size CDTb (Fig. 2*A* and Table 1). The exception was H8, which had a binding affinity of 1.9 nM. While A9, C6, and G2 had tight affinities (20–35 pM) for CDTb_D2–D3′_, they differed in their affinities for the isolated D3′ domain. The binding affinity of A9 for D3′ was 31 pM, the affinity of G2 for D3′ dropped to 180 pM, and there was no binding observed between C6 and D3′. We interpret this to mean that A9 and G2 bind to D3′, whereas C6 engages both domains 2 and 3 (Fig. 2, *B* and *C*, Tables 2 and 3). In agreement with the ELISA data, H8 and H12 bound to CDTb_D4_ (Fig, 2*D* and Table 4). In most cases, the kinetics between the Nbs and isolated domains were similar to the kinetics between the Nbs and full-sized CDTb.

**Table 1:** Summary of interactions between CDTb_D1–D4_ and its Nb binding partners

Ligand	No. of ligand densities	Mean *k*_*a*_ (M^−1^ s^−1^)	*k*_*a*_ (SD)	Mean *k*_d_ (s^−1^)	*k*_d_ (SD)	Mean *K*_D_ (M)	*K*_D_ (SD)	*R*_max_ range (RU)	Mean res (SD)	Res (SD)
A9	3	1.0 × 10^6^	5.0 × 10^5^	3.2 × 10^−5^	3.1 × 10^−5^	2.4 × 10^−11^	2.6 × 10^−11^	15.5–32.6	1.2	0.8
C6	3	7.9 × 10^5^	9.5 × 10^4^	1.4 ×10^−5^	6.3 × 10^−6^	1.8 × 10^−11^	8.1 × 10^−12^	17.7–36.6	1.0	0.3
G2	4	3.8 × 10^5^	1.2 × 10^5^	1.0 × 10^−5^	0	2.9 × 10^−11^	9.3 × 10^−12^	16.8–38.3	0.8	0.3
H8	4	5.6 × 10^5^	6.5 × 10^4^	8.9 × 10^−4^	7.4 × 10^−5^	1.6 × 10^−9^	2.3 × 10^−10^	17.8–37.2	0.6	0.1
H12	4	1.4 × 10^6^	7.2 × 10^5^	6.7 × 10^−5^	2.4 × 10^−5^	5.0 × 10^−11^	3.1 × 10^−11^	14.5–36.7	0.9	0.3

Res, residual.

**Table 2:** Summary of interactions between CDTb_D2–D3_ and its Nb binding partners

Ligand	No. of ligand densities	Mean *k*_*a*_ (M^−1^ s^−1^)	*k*_*a*_ (SD)	Mean *k*_d_ (s^−1^)	*k*_d_ (SD)	Mean *K*_D_ (M)	*K*_D_ (SD)	*R*_max_ range (RU)	Mean res (SD)	Res (SD)
A9	4	7.1 × 10^5^	1.4 × 10^5^	1.4 × 10^−5^	7.7 × 10^−6^	2.0 × 10^−11^	1.1 × 10^−11^	19.3–43.9	1.4	1.1
C6	3	1.3 × 10^6^	2.2 × 10^5^	4.4 × 10^−5^	1.7 × 10^−5^	3.5 × 10^−11^	1.5 × 10^−11^	21.1–47.7	1.0	0.2
G2	4	4.8 × 10^5^	9.6 × 10^4^	1.0 × 10^−5^	0	2.2 × 10^−11^	4.3 × 10^−12^	9.9–46.9	0.8	0.2

Res, residual.

**Table 3:** Summary of interactions between CDTb_D_ and its Nb binding partners

Ligand	No. of ligand densities	Mean *k*_*a*_ (M^−1^ s^−1^)	*k*_*a*_ (SD)	Mean *k*_d_ (s^−1^)	*k*_d_ (SD)	Mean *K*_D_ (M)	*K*_D_ (SD)	*R*_max_ range (RU)	Mean res (SD)	Res (SD)
A9	3	3.3 × 10^5^	5.9 × 10^4^	1.0 × 10^−5^	0	3.1 × 10^-11^	5.7 × 10^−12^	15.4–40.1	0.7	0.2
G2	4	2.4 × 10^5^	3.2 × 10^4^	4.1 × 10^−5^	3.1 × 10^−5^	1.8 × 10^-10^	1.4 × 10^−10^	11.9–45.1	0.7	0.3

Res, residual.

**Table 4:** Summary of interactions between CDTb_D4_ and its Nb binding partners

Ligand	No. of ligand densities	Mean *k*_*a*_ (M^−1^ s^−1^)	*k*_*a*_ (SD)	Mean *k*_*d*_ (s^−1^)	*k*_d_ (SD)	Mean *K*_D_ (M)	*K*_D_ (SD)	*R*_max_ range (RU)	Mean res (SD)	Res (SD)
H8	4	1.4 × 10^6^	4.2 × 10^5^	8.3 × 10^−4^	6.3 × 10^−5^	6.3 × 10^−10^	2.0 × 10^−10^	8.9–27.0	0.5	0.1
H12	4	3.1 × 10^6^	4.2 × 10^5^	1.3 × 10^−4^	3.4 × 10^−5^	4.2 × 10^−11^	1.2 × 10^−11^	19.1–54.8	1.4	0.7

*k*_*a*_, association rate constant; *k*_d_, dissociation rate constant; *K*_D_, equilibrium dissociation constant; Res, residual; *R*_max_, parameter reflecting SPR signal at binding saturation as determined in the kinetic fit.

### Epitope binning of Nbs confirmed three distinct CDTb epitopes that can be utilized in quantitative ELISA

**Figure 3:**
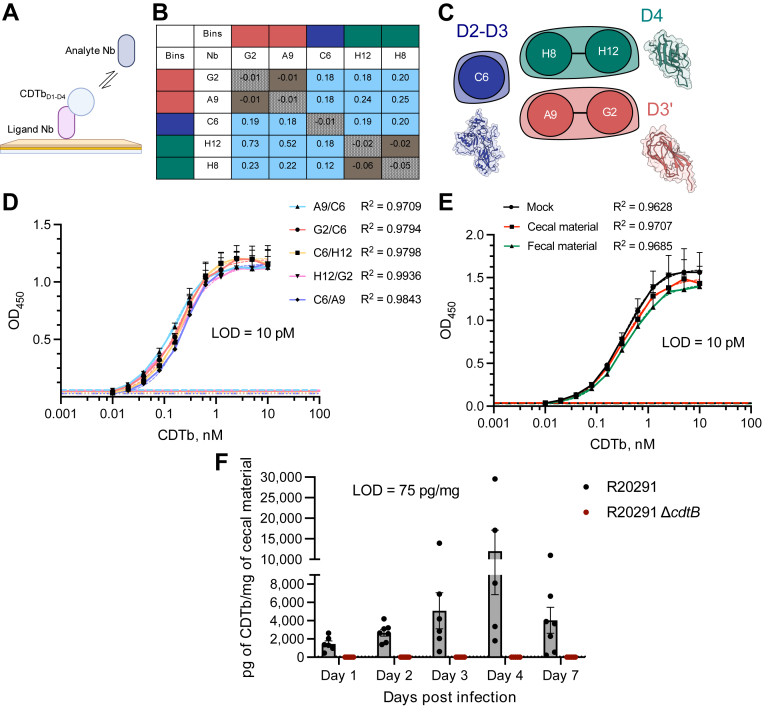
**Epitope binning of nanobodies (Nbs) confirmed three distinct CDTb epitopes that can be utilized in quantitative ELISA.***A,* schematic of the epitope binning assay. The figure was created with BioRender.com using a premium institutional license. *B,* heat map showing blocking relationships of analyte–ligand Nb pairs. *Brown squares* represent competition between the corresponding analyte and ligand, and *light blue squares* indicate “sandwiching” or no competition. The competition threshold was set to 0.05 to 0.1. *Shaded brown squares* along the diagonal represent self–self interactions. These were confirmed to be blocking in both orientations. *C,* network plot showing that Nbs were grouped into three distinct epitope bins—bin 1 (*red*—A9 and G2), bin 2 (*green*—C6), and bin 3 *(blue*, H8 and H12). Nbs are represented as nodes, blocking relationships are represented as chords, and epitope bins are represented as envelopes. *D,* CDTb-binding Nb pairs (capture/detection) used to generate quantitative ELISA assays. Five Nb pairs show good agreement with the CDTb limit of detection (LOD) of 10 pM (shown for each pair in *color-matched lines* at the bottom of the *Y*-axis). G2–C6 pair (in *bold*) was used for downstream analyses. *E,* standard curves in uninfected mouse cecal and fecal materials diluted to 10 mg/ml in PBS-T–BSA with spiked in titration of purified CDTb. Neither background changed the LOD compared with the mock condition (PBS-T–BSA only). In both (*D*) and (*E*), standard curves were constructed by interpolating the data using a sigmoidal four-parameter logistic (4-PL) curve fit. Data points are connected by *solid lines*, and color-matched curve fits are shown in *dashed lines*. Each data point represents the mean ± SD of three independent biological experiments (n = 3). Each biological experiment consisted of two technical replicates, and the average value of both technical replicates was used. *F,* CDTb was quantified in cecal material (diluted to 10 mg/ml in PBS-T–BSA) of euthanized mice during the indicated timepoints of R20291 or R20291 Δ*cdtB* infections. Bars represent mean ± SEM of the group; dots represent an individual mouse within the group. Number of animals: day 1—n = 6 (R20291), n = 6 (R20291 Δ*cdtB*); day 2—n = 7 (R20291), n = 7 (R20291 Δ*cdtB*); day 3—n = 6 (R20291), n = 6 (R20291 Δ*cdtB*); day 4—n = 5 (R20291), n = 5 (R20291 Δ*cdtB*); and day 7—n = 7 (R20291), n = 5 (R20291 Δ*cdtB*). *In vivo* experiments were independently performed two times (with three to four animals per group). BSA, bovine serum albumin; CDTb, *C*los*tridioides difficile* transferase toxin, B subunit; PBS-T, PBS with Tween-20.

The Carterra LSA^XT^ system also supports competitive epitope-binning workflows to determine whether two antibodies recognize the same antigenic epitope. The assay begins with the preparation of a covalently coupled antibody array. Each antibody is then injected individually as the analyte, together with a constant concentration of antigen. After each injection, the array surface is regenerated to remove bound analyte before the next cycle. Two competitive-binning formats are available depending on valency of the antigen: classical format, sequential injections of antigen followed by antibody, is used for monomeric antigens, whereas the Premix format, a single injection of antigen precomplexed with a saturating concentration of antibody, is used for multimeric antigens. In our sandwich assay, Nbs were tested for binding to monomeric CDTb_D1–D4_ that was first captured *via* immobilized Nbs (Fig. 3*A*). As a result, Nbs were tested in a pairwise combinatorial manner, and those that competed for the same binding region were grouped together into bins. The analysis revealed three distinct bins—A9 and G2 in bin 1, C6 in bin 2, and H8 and H12 in bin 3 (Figs. 3, *B* and *C* and S2). Classification of Nbs based on epitope diversity agreed with the initial assignment of domain specificity (Fig. 1*D*) and the kinetics binding data (Fig. 2).

We then used the high-affinity Nbs against distinct CDTb domains to develop a Nb-based sandwich ELISA with the goal of quantifying CDTb in physiological samples. Briefly, 96-well plates were first coated with nonbiotinylated “capture” Nbs. Then, CDTb (recombinant protein in regular diluent or spiked into an uninfected matrix of interest or native protein in infectious physiological samples) was bound by the capture Nb, followed by the addition of biotinylated “detection” Nb. Incubation with streptavidin conjugated to horseradish peroxidase allowed the sandwiched complex to be detected by the substrate.

We narrowed the Nb pairings down to five effective pairs that detected purified activated monomeric CDTb with a limit of detection (LOD) of 10 pM, the lowest concentration at which the signal was above the negative control (Fig. 3*D*). In these pairings, two Nbs from different epitope bins were used, for instance, A9 (D3′ specific) in conjunction with C6 (D2–D3 specific). While all Nbs detected CDTb relatively well, we chose the G2/C6 (capture/detection) pair for downstream analysis. The signal specificity was confirmed when each component of the sandwich ELISA was omitted one at a time, resulting in no signal (Fig. S3*A*).

Next, we tested whether purified CDTb can be detected when added to a complex matrix such as uninfected cecal and fecal contents of mice. Our goal was to determine if standard curves in these backgrounds differ from the standard curve generated in the diluent only (PBS with Tween-20 [PBS-T]–bovine serum albumin [BSA]). When resuspended, uninfected cecal and fecal materials were diluted in PBS-T–BSA to 10 mg/ml and supplemented with serial dilutions of purified CDTb; the LOD did not change (Fig. 3*E*). In addition, when fit values of the standard curve in PBS-T–BSA were compared with the ones from the standard curve in cecal contents, there was no significant difference between the curves (Fig. S3*B*). We thus decided to dilute cecal content samples from infected mice to 10 mg/ml and include standard curves in PBS-T–BSA on each plate when quantifying CDTb during *in vivo* infection.

Finally, we quantified CDTb in the cecal contents of mice infected with R20291 (TcdA^+^TcdB^+^CDTa^+^CDTb^+^) and R20291 *ΔcdtB* (TcdA^+^TcdB^+^CDTa^+^CDTb^-^). While the toxin subunit was successfully detected over the time course of R20291 infection, the signal was absent or below the LOD during R20291 *ΔcdtB* infection, further confirming the specificity of the assay for native CDTb protein (Fig. 3*F*). The levels of CDTb started to accumulate in the cecum during the first 2 days, with average concentrations of 1460 pg/mg and 2670 pg/mg, on days 1 and 2, respectively. These concentrations peaked on days 3 and 4 with averages of 5090 pg/mg and 11,970 pg/mg, respectively, and dropped back to 4040 pg/mg at 7 days.

We recognized that since Nb C6 binds to CDTb within the D2–D3 oligomerization interface, the G2–C6 Nb pair used for CDTb quantification in cecal material may not have captured oligomeric states of CDTb because exposed D2–D3 epitopes within CDTb monomer could be occluded within CDTb heptamer. To investigate this further, we used the A9–H12 Nb pair that recognizes D3′ and D4 epitopes, which would increase the possibility of detecting oligomeric CDTb. While the A9–H12 pair had a slightly lower dynamic range for detecting purified monomeric CDTb, it recognized heptameric CDTb significantly better than the G2–C6 pair, indicating that Nb C6, indeed, does not recognize oligomers efficiently (Fig. S4, *A* and *B*). Although some signal was detected by the G2–C6 pair, it most likely corresponds to monomeric CDTb within the heptameric sample, possibly because of some disassociation of heptamers into monomers over time. We then wondered if the use of the G2–C6 pair for the detection of CDTb in the cecal material had led us to miss the quantitation of CDTb heptamers. We therefore used the A9–H12 pair to quantify CDTb in the cecal contents of five mice 4 days post R20291 infection. Levels of detected CDTb were comparable between both pairs (Fig. S4*C*), indicating that monomers constitute the majority of CDTb within the cecal contents of mice during R20291 infection.

### High neutralization potency of anti-CDTb Nbs

**Figure 4:**
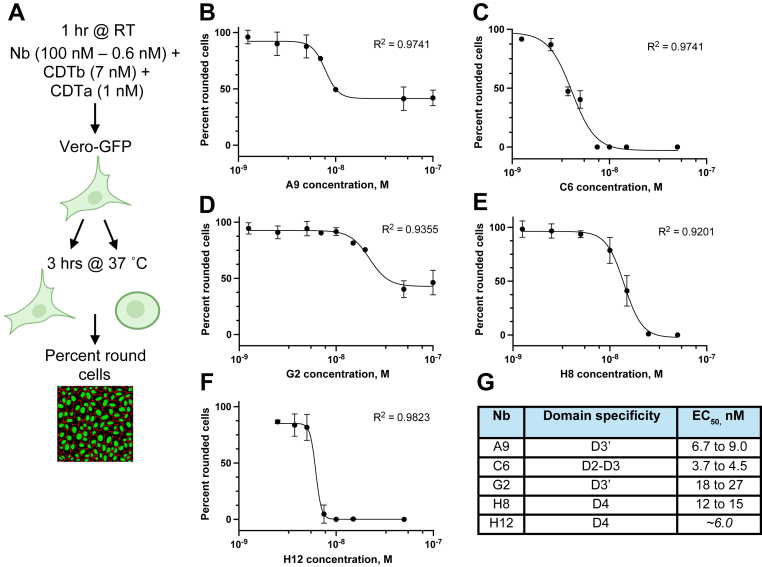
**High neutralization potency of anti-CDTb nanobodies (Nbs).***A,* schematic of the neutralization assay in Vero-GFP cells. The figure was created with BioRender.com using a premium institutional license. The concentrations of purified CDTb and CDTa were kept constant (7 nM of activated monomer and 1 nM, respectively), and Nbs were titrated according to the graphs. Cell rounding curves depict the protective effects of (*B*) A9, (*C*) C6, (*D*) G2, (*E*) H8, and (*F*) H12 Nbs. *G,* neutralization data summarized by EC_50_ values obtained *via* a nonlinear fit to the (agonist) *versus* response variable slope (four parameters) model (GraphPad Prism). Each data point represents the mean ± SD of three independent biological experiments at a 3-h postintoxication timepoint (n = 3). Each biological experiment consisted of two technical replicates, and the average value of both technical replicates was used. For H12 (*F*), the analysis did not deduce EC_50_ value (most likely because of a sharp change in percent rounding between the two reported concentrations). Thus, an approximate value of 6.0 nM was manually assigned based on the curve fit. CDTa, *Clostridioides difficile* transferase toxin, A subunit; CDTb, *Clostridioides difficile* transferase toxin, B subunit.

We then tested the Nbs for their capacity to neutralize the cytopathic cell rounding of epithelial Vero-GFP cells by purified CDT (1 nM of CDTa and 7 nM of activated monomeric CDTb combined) (Figs. 4*A* and S5). All five Nbs showed potent protective effects characterized by EC_50_ values of 3 to 30 nM at 3 h postintoxication (Fig. 4, *B*–*F*). Nearly all Nbs were able to block cell rounding at an equimolar ratio with monomeric CDTb (Fig. 4*G*). The relative efficiency of neutralization did not depend on which CDTb domain is targeted. For instance, C6, H12, and A9 were the most potent neutralizers and target three different domains (D1–D3, D4, and D3′, respectively). H8 and H12, both directed against D4, differed in cell rounding inhibition by ∼2-fold, and A9 and G2, both directed against D3′, by ∼2 to 4 fold. At 8 h postintoxication, C6 and H12 Nbs retained their neutralization properties at equimolar concentrations (Fig. S6). Notably, Nbs A9 and G2 lost their protective abilities, even at the highest concentrations.

### Functional *in vitro* assays support predicted mechanisms of neutralization

We then turned to structural prediction to better define the neutralization epitopes. We tested whether AlphaFold3 could yield high-confidence prediction models and discovered that four of five CDTb–Nb models (Figs. S7, *A*–*D* and S8) were generated with predicted template modeling (pTM) scores above 0.5 and interface pTM scores above 0.8, suggesting a high accuracy of predictions (32). The CDTb + H8 complex was the only model with poor scores (interface pTM = 0.13 and pTM = 0.63), and we decided not to characterize this complex further. Consistent with the kinetics and epitope binning data, A9 and G2 interacted with D3′, C6 interacted with D2, and H12 interacted with D4 domains. While AlphaFold3 has impressive performance in the accurate placement and orientation of proteins involved in biomolecular interactions, it still has limitations in the accuracy of interface packing contacts (32). The AlphaFold3 models should not be interpreted as high-resolution structures. While we do not consider these models accurate enough to describe protein–protein interactions on the atomic resolution level, we found them useful for initiating rational hypotheses regarding how binding of Nbs to one CDTb monomer could affect the rest of the protomers within the heptameric prepore. For example, binding of A9 and G2 to D3′ occurs at the outer ridge of the domain, which does not interfere with the oligomerization interface. On the other hand, binding of C6 to the D2 domain of CDTb monomer leads to clashes with adjacent protomers when this interaction is mapped into the assembled CDTb heptamer. Last, H12 does not directly contact the CDTb oligomerization interface, but it may interfere with receptor binding.

According to our AlphaFold3 models, the C6 and H12 Nbs engage CDTb epitopes *via* the elongated CDR3 loop (Fig. S7*B* and S7*D*), a feature commonly associated with epitope recognition within Nb paratopes (29). On the contrary, the models suggest that the A9 and G2 Nbs face and contact CDTb *via* a noncanonical binding mode with interactions between two D3′ β-strands and antiparallel Nb β-strands (Fig. S7, *E* and *F*). Interestingly, several studies have shown Nb–antigen interactions that are mediated by residues from Nb framework regions (33, 34, 35). We utilized chemical crosslinking mass spectrometry (MS) to identify crosslinked lysine residues within the expected D3′–Nb proximity. For the CDTb + A9 complex, two crosslinks were identified, and when the interactions were mapped onto the AlphaFold3 model, they were within the recommended maximum distance cutoff of 26 to 30 Å for BS3 crosslinker (Figs. S7*E*, S9, *A* and *B*, and Table S3) (36). For the CDTb + G2 complex, four crosslinks were identified (Figs. S7*F*, S9, *C*–*G*, and Table S4). Overall, these crosslinks support β-sheet protein–protein interactions between D3′ of CDTb and the A9 and G2 Nbs.

**Figure 5:**
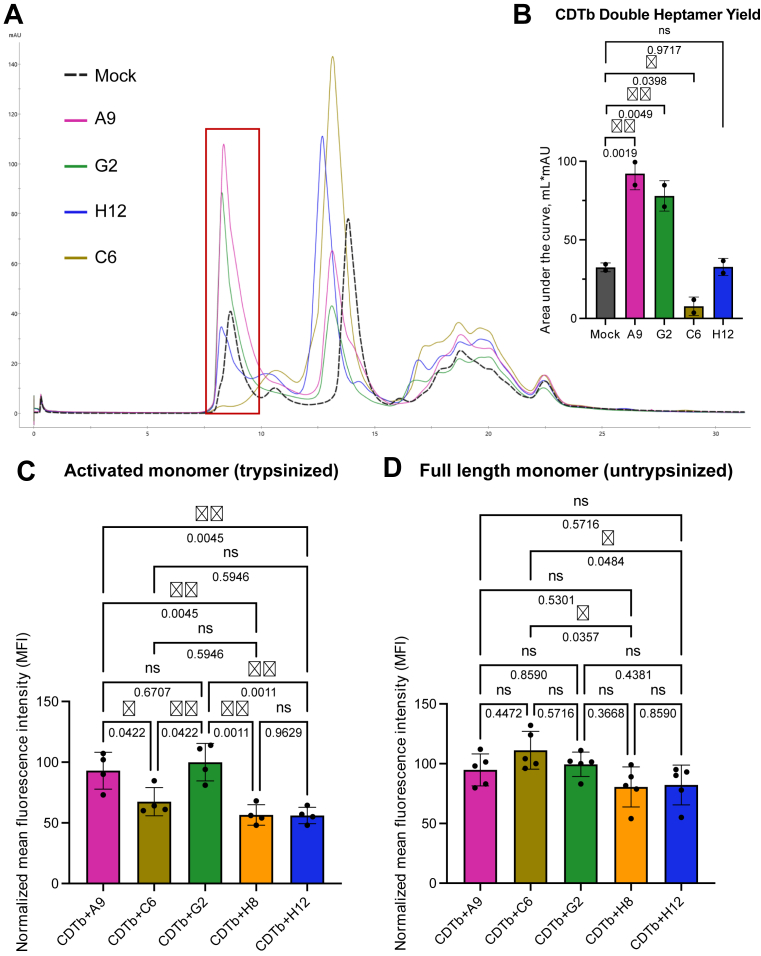
**Functional *in vitro* assays support predicted mechanisms of neutralization.***A,* oligomerization assay showing that C6 prevents whereas A9 and G2 promote CDTb oligomerization *in vitro*. Representative overlayed size-exclusion chromatography (SEC) profiles of full-length (pro) CDTb trypsinized with or without nanobodies (Nbs). The *red box* shows a double heptamer peak, which is the readout of CDTb oligomerization. About 1.5 mg of full-length (pro) CDTb at 7.9 mg/ml final concentration was trypsinized for each run. Each nanobody was added in 5X molar excess relative to CDTb. *B,* quantification of the area under the curve of the double heptamer peak. Bars represent mean ± SD of the group; dots represent an individual independent biological experiment. One-way ANOVA with Holm–Šídák’s multiple comparisons test was used to calculate statistical significance. The experiment was independently performed two times (n = 2). Each biological experiment consisted of one technical replicate. Quantification of Vero cells bound by (*C*) trypsinized CDTb-Alexa 647 or (*D*) full-length (pro) CDTb-Alexa 647 in the presence of Nbs using flow cytometry. Mean fluorescence intensity (MFI) of Alexa-647^+^ live cells is reported relative to CDTb treatment without the Nbs (treated as 100). About 25 nM of CDTb preincubated with 100 nM of Nbs (4X excess) were applied to the cells. Bars represent mean ± SD of the group; dots represent an individual independent biological experiment. One-way ANOVA with Holm–Šídák’s multiple comparisons test was used to calculate statistical significance. For trypsinized CDTb, the experiment was independently performed four times (n = 4). For full-length (pro) CDTb, the experiment was independently performed five times (n = 5). Each biological experiment consisted of one technical replicate. CDTb, *Clostridioides difficile* transferase toxin, B subunit.

Finally, we decided to collect experimental functional data to support the predicted neutralization mechanisms based on the obtained CDTb–Nb models. To test the hypothesis that C6 prevents CDTb oligomerization, we performed an oligomerization assay by preincubating the Nbs with full-length (pro-CDTb) monomer and subjecting the complex to trypsinization *in vitro*. In addition to activated monomer, the trypsinization of CDTb *in vitro* leads to the formation of a dimer of heptamers—one in the pore state and one in the noninserted prepore state (14). This double heptamer can be separated as a high-molecular weight fraction by size-exclusion chromatography, which was the main readout of oligomerization. Trypsinization in the presence of C6 completely prevented the formation of CDTb double heptamer (Figs. 5, *A* and *B* and S10). Consistent with the lack of oligomers, most of CDTb remained in an activated monomer state, as suggested by the peak that is doubled in size and shifted in molecular weight because of its binding to the Nb. Interestingly, the double heptamer peak obtained during trypsinization with A9 and G2 was doubled in size compared with the double heptamer peak of CDTb alone. Our interpretation is that binding of A9 and G2 may improve the solubility of CDTb oligomers, resulting in a higher yield of double heptamers. When the D4–H12 interaction was modeled within the CDTb heptamer, we concluded that H12 does not directly contact a CDTb oligomerization interface. Consistent with our interpretation, the CDTb double heptamer peak following trypsinization in the presence of H12 was comparable to the peak of CDTb trypsinized by itself. Overall, this assay supports the idea that binding of Nb C6 to one CDTb monomer can interfere with the stepwise addition of the other CDTb protomers in the context of CDTb heptamer, which prevents formation of functional CDTb heptamers necessary for CDT toxicity.

To test whether inhibition of cell binding explains neutralizing properties of H12 and H8 Nbs, we utilized a flow cytometry–based approach in which the preincubated complex of fluorescently labeled CDTb and the 4X molar excess of Nbs was added to Vero cells in suspension and incubated at 37 °C. At first, we used trypsin-activated CDTb monomer to allow for heptamer formation at the cell surface, which would amplify fluorescent signal readout. As expected, Nbs H12 and H8 led to a significant reduction of CDTb attached to the cell surface (Figs. 5*C*, S11, and S12). D3′-specific Nbs A9 and G2 did not affect CDTb binding to the cell surface under these experimental conditions. Unexpectedly, we observed that the Nb C6 also reduced CDTb binding to the cell surface. We recognized that activated CDTb monomers bound by C6 are most likely still capable of receptor binding. However, because the Nb prevents CDTb heptamer formation, the signal at the cell surface does not get amplified, leading to a lower intensity. Thus, our interpretation is that the signal from CDTb monomer–C6 complex binding to the cell surface leads to a similar readout as CDTb heptamer–H8/H12 complex, uncapable of engaging the cell surface. To confirm this explanation and discern these phenotypes further, we utilized fluorescently labeled full-length pro-CDTb monomer to block the ability of CDTb to form heptamers, which would eliminate this variable across all treatments. As expected, the dynamic range of signal intensity decreased dramatically in this setup, and H8- and H12-dependent differences were seen but not as strikingly as with the activated monomer (Figs. 5*D*, S11, and S12). However, C6 did not lead to any reduction of CDTb binding to the cell surface, and, in fact, this Nb had the lowest effect compared with the rest of the Nbs, especially H8 and H12.

Overall, these data agree with functional predictions based on epitope mapping from AlphaFold3 models in the context of CDTb heptamer. We conclude that Nb C6 neutralizes CDT cytotoxicity by preventing heptamer formation, whereas Nbs H8 and H12 neutralize CDT by interfering with cell surface binding.

## Discussion

In the current study, we sought to create a toolkit for toxin neutralization and quantitation in physiological samples, as such reagents are limited and could advance our knowledge of the mechanisms of intoxication and disease caused by CDT. We created a Nb library from an alpaca vaccinated with CDTb and screened a panel of 94 isolates for binding to defined CDTb structural domains. We focused on five clones for downstream characterization: C6 that binds the D2–D3 domains responsible for CDTb oligomerization and pore formation, A9 and G2 that bind the D3′ domain, and H8 and H12, which bind the D4 domain responsible for receptor binding. The SPR studies confirmed the domain specificity assignments and further revealed tight binding of the Nbs to CDTb. Each of the Nbs was shown to be a potent neutralizer of CDT-induced cell rounding. We used AlphaFold3 to generate structural models of the Nb–CDTb complexes and hypotheses about the mechanism of neutralization. These models were then corroborated with crosslinking studies and *in vitro* functional assays. For example, Nb C6 was predicted to bind the oligomerization interface within the D2 domain. The oligomerization of CDTb is required for CDTa binding and the CDTb-mediated delivery of the CDTa enzyme into the cytosol of the host. We expected CDTb monomers to bind the cell surface in the presence of C6 but that CDTb heptamers would not. Our flow cytometry experiments were consistent with this hypothesis. In the case of H8 and H12, we realized that while binding of these Nbs may allow all seven CDTb protomers to come together and form a heptamer, it will certainly block the epitopes necessary for cell surface binding. Consistent with this, impaired binding was observed in the flow cytometry experiments. The mechanism of neutralization by D3′-specific A9 and G2 Nbs remains to be investigated and presents a challenge, given that the role of D3′ during intoxication is not well understood. Notably, neither Nb provided full protection, even at the highest concentrations, and the neutralization curves were distinct from those of the other neutralizing Nbs at 3 h postintoxication. We note that after looking at the neutralization data at the 8-h endpoint, the partial protection was completely gone. Our interpretation is that these Nbs affect the kinetics of toxin binding. It may also indicate that CDTb uses bimodal binding in which D3′ localizes CDTb (either closer or faster) to the cell surface without being required for receptor binding and toxin internalization. In addition, the A9 and G2 Nbs are predicted to interact with CDTb in a noncanonical way that does not primarily rely on the CDR3 loop. Instead, they stacked “in parallel” to the D3′ domain *via* β-sheet interactions. The accuracy of the predicted AlphaFold3 models needs to be further assessed in experimental structural studies.

The neutralization studies in Vero-GFP cells revealed that each of the five selected Nbs protected the cells from CDT-induced rounding. A9, C6, and H12 were able to do so at nearly equimolar concentrations. Effective equimolar inhibition of binary toxin–induced cytotoxicity by Nbs was also observed by Unger *et al.* (37), who similarly characterized several Nbs against CDTa and CDTb from phage display libraries generated from immunized llamas. In this study, the team reported three CDTa-specific and two CDTb-specific Nbs that neutralized the cytotoxicity of CDT in human colonic epithelial HT29 cells; one CDTa directed and one CDTb directed were able to do so at equimolar concentrations. In agreement with our data, the study proposed that the CDTb-specific Nbs could block either heptamer assembly or interactions of CDTb with CDTa or lipolysis-stimulated lipoprotein receptor. A more recent study isolated two CDTb-specific neutralizing monoclonal antibodies that protected HT29 cells from death in a concentration-dependent manner (38). The study further used electron microscopy and X-ray crystallography to identify the neutralizing epitopes: first at the D4 oligomerization interface and second at the D3 and D3′ interface. Although the first antibody directly interacts with D4 at the oligomerization interface, contacting D4 of the adjacent protomer within the assembled CDTb oligomer, the second antibody does not directly contact adjacent protomers; yet it still prevents CDTb heptamer formation because of a steric clash.

The successful isolation of Nbs recognizing discrete epitopes encouraged us to establish a sandwich ELISA for CDTb because one hurdle in understanding the impact of CDT has been a lack of validated ELISA reagents for quantifying CDT levels *in vivo.* We identified five Nb pairs that are highly sensitive *in vitro* ELISA reagents. The sensitivity levels were further showcased when purified CDTb was detected at the same LOD in complex mixtures, such as mouse cecal content and feces, diluted ∼100 fold. This enabled us to process infected cecal samples and circumvent potential interference of the complex environment with toxin detection. Finally, we monitored the dynamics of CDTb production over 5 days post R20291 infection, as there is a lack of data on the levels and timing of toxin concentrations in experimental models of infection. CDTb was detected as early as 1 day and as late as 7 days postinfection. Despite higher datapoint variance, the levels of CDTb spike during days 3 and 4. This is consistent with TcdA and TcdB quantifications conducted in our earlier study (39), suggesting that overall toxin production may occur synergistically in the R20291 background. We hope the time-course quantification is beneficial for those designing *in vitro* experiments relying on the knowledge of physiological toxin concentrations during infection. The reported ELISA reagents could also be developed for toxin detection and diagnostic purposes in a clinical setting.

Limited data exist on the quantification of CDT in physiological samples or during animal infections. Camren *et al.* (26) were the first to detect CDT in feces from *C. difficile* patients. The study reported that CDTb was found in 19 of 36 (53%) fecal samples assayed from patients colonized with *cdt*^+^ isolates. Fecal samples that were CDTb positive by ELISA contained 22 to 1325 ng CDTb/ml. Recently, Meza-Torres *et al.* (25) used commercial anti-CDTb antibodies in ELISA format to measure CDT levels in feces from mice infected with UK1 TcdA^-^TcdB^-^CDT^+^, an isogenic mutant of *C. difficile* UK1 epidemic strain (NAP1/BI/ribotype 027). Unlike the R20291 background in which CDTb was detected at 1 day postinfection, CDTb started being produced at 7 days postinfection in the UK1 background, a much later time point. The concentrations were increasing up to 13 days postinfection (from ∼3 to 27 ng/ml), correlating with CDT-induced changes in mucin thickness, decreased number of goblet cells, and increased fecal lipocalin-2 levels sustained throughout the infection. This further exemplifies the well-established idea in the field that toxin expression and secretion varies across *C. difficile* strains *in vitro* and *in vivo* (31, 40, 41). Evaluation of CDT levels produced from different strains *in vitro* and *in vivo* merits further investigation. The knowledge of distinct epitopes remains advantageous when pursuing antitoxin therapies where simultaneous targeting of more than one functionally important epitope is needed.

## Experimental procedures

### Ethics statement

This study was approved by the Institutional Animal Care and Use Committee at the Vanderbilt University Medical Center. Our laboratory animal facility is accredited to the Association for Assessment and Accreditation of Laboratory Animal Care International and adheres to the guidelines described in the Guide for the Care and Use of Laboratory Animals. Prior to antibiotic treatment, mice were assimilated to the new facility for 1 week to reduce stress. Mice were housed in a pathogen-free room with 12-h cycles of light and dark, clean bedding, and free access to food and water. Cages were changed every 2 weeks. The health of the mice was monitored daily, and severely moribund animals were humanely euthanized. Immunization of alpacas at the Turkey Creek Biotechnology was done in accordance with Turkey Creek Biotechnology Institutional Animal Care and Use Committee.

### Isolation of anti-CDTb Nbs

Recombinant CDTb (1 mg/ml) was used to immunize an alpaca, Royal Margarita, with 150 to 300 μg of CDTb mixed 1:1 by volume in Gerbu adjuvant. Eight additional immunizations were done on days 13, 27, 42, 56, 125, 139, 153, and 174. Blood was drawn on day 181, and PBMCs were isolated from 17 ml of blood. A complementary DNA library was constructed from 8.25 μg of total PBMC RNA using oligo dT primers and Superscript IV reverse transcriptase (ThermoFisher). Two rounds of PCR were used to isolate VHH-encoding DNA and separate it from Vh-encoding DNA as described (31). The gel-purified PCR fragments were cloned into a pADL22 (Antibody Design Labs)-derived vector with an N-terminal PelB leader sequence and C-terminal His tag and hemagglutinin (HA) tag, and a phage library was constructed following the manufacturer’s (Antibody Design Lab) protocols. A single round of panning was done against 2 μg of CDTb immobilized in a well of a MaxiSorb plate (ThermoFisher). Bound phages were eluted with 200 μl of 100 mM (pH 2.2) glycine, neutralized with 100 μl of 1 M Tris–HCl (pH 8), allowed to infect TG1 cells, and stored as a glycerol stock. This stock was diluted, and single colonies were isolated and grown in 1 ml cultures in deep-well 96 blocks. Expression was induced with 1 mM IPTG, grown overnight at 28 °C, and harvested by centrifugation. Nb was released by two freeze–thaw cycles in PBS. Positive clones were identified by ELISA using MaxiSorb plates coated with 1 μg/well CDTb, crude lysates as the Nb source, HA–horseradish peroxidase (HRP) (ThermoFisher), and one step of 3,3′,5,5′-tetramethylbenzidine substrate (ThermoFisher).

### CDTb domain specificity assignment (domain screen)

Protein lysates were tested for their binding properties to CDTb_D1–D3_ (residues 43–615), CDTb_D3’_ (residues 616–750), and CDTb_D4_ (residues 760–876) by ELISA. Briefly, 500 ng/well of CDTb constructs were immobilized in a 96-well plate at 4 °C overnight. The next day, the plates were washed four times with PBS-T wash buffer (PBS supplemented with 0.125% Tween-20) and incubated with blocking buffer (PBS-T + 2% BSA), followed by four washes with wash buffer. Protein lysates were added to the coated plate, and upon incubation, the plates were washed four times with wash buffer. Next, anti-HA antibody (1:2000 dilution) was added to the plates and incubated for 90 min at room temperature. After the plates were washed four times with wash buffer, anti-mouse HRP (1:2000 dilution) was added to the plates and incubated for 1 h at room temperature. The plates were then washed four times with wash buffer, and 75 μl/well of one step of UltraTMB ELISA substrate solution (Thermo Fisher, 34029; equilibrated to room temperature) was added to each well. Finally, reactions were quenched with 2 M H_2_SO_4_ in 5 min, and absorbance was measured at 450 nm. The signal from negative controls for each domain was averaged, multiplied by two, and set as a threshold. If the signal value for an Nb clone was above the threshold, it was assigned as a positive domain binder in Figure 1. The model of monomeric CDTb structure showcasing the domains was obtained from the Protein Data Bank (ID: 6O2N). The figure was made in UCSF ChimeraX.

### Nb sequence analysis

The DNA plasmids containing Nb clones were sequenced (Azenta). Alignments of full amino acid sequences were made in CLUSTAL OMEGA (42). Phylogenetic analysis was performed with RAxML using the raxmlGUI platform (43). Results were visualized in ITOL, version 6.9.1 (44).

### Nb expression and purification

Selected Nb sequences were cloned into the pc.DNA3.4 vector using the following cloning sites: 5′ AgeI and 3′ EcoRV (GenScript). Plasmid DNA was propagated in DH5α-competent *E. coli* cells and purified (Qiagen MaxiPrep). Nbs were expressed and purified from Expi293F mammalian cells (Thermo Fisher). Specifically, the cells were seeded at 1 × 10^6^ cells/ml the day before transfection. On the day of transfection, DNA was diluted to 1 μg/ml of cell culture in OPTI-MEM (Gibco; 11058021), and transfection was performed at 2.3:1 ratio of polyethylenimine (PEI) to DNA. PEI was also diluted in OPTI-MEM and incubated for 10 min at room temperature. PEI was then combined with diluted DNA, and the complex was gently mixed and incubated for 15 min at room temperature before addition to the cells. The cells were then grown at 37 °C with 8% CO_2_ for 7 days. On day 7 post-transfection, the cellular supernatant was harvested and centrifuged. The supernatant was then filtered (0.22 μM), and binding buffer (50 mM NaPi [pH 8.0], 300 mM NaCl) was added to it until the pH reached ∼7.6. 3 ml of TALON Superflow resin (Cytiva) per every 220 ml of cellular culture was equilibrated with binding buffer, added to the supernatant, and the mixture was incubated at 4 °C for 1 h with agitation. The mixture was added to a gravity column and allowed to flow through. The resin was washed with ∼20 column volumes of wash buffer (25 mM NaPi [pH 7.0], 500 mM NaCl) and eluted with ∼10 column volumes of elution buffer (25 mM NaPi [pH 7.0], 500 mM NaCl, 200 mM imidazole [pH 7.0]). The elution fraction was concentrated in an Amicon centrifugal spin concentrator (3 K molecular weight cutoff), allowed to buffer exchange into 25 mM NaPi (pH 7.0), 500 mM NaCl, sterile filtered, and flash frozen in liquid nitrogen.

To biotinylate Nbs, we used biotin ligase (BirA). pET21a-BirA was a gift from Alice Ting (Addgene plasmid #20857; http://n2t.net/addgene:20857; Research Resource Identifier: Addgene_20857), and the enzyme was prepared as previously described (45). Briefly, purified Nbs were dialyzed into 20 mM NaPi (pH 7.0) and 150 mM NaCl buffer to lower salt concentration. The Nbs at concentrations between 0.7 and 1 mg/ml were combined with the following reagents to set up biotinylation reactions: 10 mM ATP, 10 mM MgAcO, 100 μM biotin, 1:10 mg BirA:Nb (final solution). The reactions proceeded overnight at 4 °Cwithout agitation. The final product was concentrated down to 1 ml and applied to an S200 Increase 10/300 GL column equilibrated with 20 mM Tris (pH 7.0), 500 mM NaCl buffer. Fractions of interest were pooled together, sterile-filtered, and flash frozen in liquid nitrogen. After every purification step, protein fractions were run on an SDS-PAGE gel to assess protein purity.

### CDTb toxin constructs expression and purification

Constructs and their purification schemes are listed in Table 5. To label CDTb for flow cytometry experiments, CDTb was concentrated at least to 2 mg/ml and incubated with Alexa Fluor 647 NHS ester (Thermo; A20006) at 10X molar excess for 1 h at room temperature with occasional pipetting protected from light. The reaction was quenched with 10 mM Tris (pH 8.0) and applied to an S200 column in 20 mM Hepes (pH 8.0), 100 mM NaCl to remove unreacted dye. On average, labeling ratios were two to three fluorophores per CDTb monomer.

**Table 5:** Toxin protein constructs used in the study

Construct	Lab code	Purification notes
CDTb_D1–D4_	pBL870	Same construct and purification as in (14, 39).
CDTb_D1–D3_	pBL1318	Same purification scheme as for CDTb_D1–D4_
CDTb_D2–D3′_	pBL962	Same purification scheme as for CDTb_D1–D4_
CDTb_D3′_	pBL915	Same construct and purification as in (14)
CDTb_D4_	pBL914	The clone was made by mutagenizing pBL870 parent plasmid. In this clone, 6x His is located on C terminus. The protein was expressed and purified as CDTb_D1–D4_ with the following exception. After nickel column, the elution fraction was concentrated to 1 ml and applied to an S75 column in 20 mM Hepes (pH 8.0), 100 mM NaCl buffer. Fractions of interest were flash frozen
CDTa	pBL926	Same construct as in (14, 39). Same purification scheme as in (39)

### *In vitro* toxin neutralization assays

Vero-GFP cells were produced as described previously (39). The cells were maintained in Dulbecco's modified Eagle's medium (DMEM) + 10% heat-inactivated fetal bovine serum (FBS) and puromycin (10 μg/ml final) and cultured at 37 °C with 5% CO_2_. One day prior to the assay, cells were seeded into a 96-well plate at 24,000 cells per well and allowed to grow overnight in DMEM + 10% FBS without antibiotics. On the day of the assay, purified CDTb and CDTa were incubated with serial dilutions of Nbs for 1 h at room temperature and then added to the cells (final CDT concentration is 7 nM CDTb and 1 nM CDTa; final Nb concentration range is 100–0.6 nM). GFP images were taken every 30 min on the BioTek Cytation 5 plate reader. From these images, the total number of rounded and nonrounded cells was counted as described (46). The percent of rounded cells was determined using the following formula: $\text{Percent}\;\text{of}\;\text{rounded}\;\text{cells}=\frac{R\left(t\right)-R\left(0\right)}{T\left(t\right)-R\left(0\right)}$, where *R*(*t*) = total number of round cells at time point *t*; *R*(0) = total number of round cells at time point 0; and *T*(*t*) = total number of cells at time point *t*. Percent rounded values were then normalized to the toxin-only–treated cell value. Data are shown at a 3-h postintoxication time point. EC_50_ was calculated in GraphPad Prism (GraphPad Software, Inc) by the (inhibitor) *versus* response - variable slope (four parameters) model.

### SPR binding kinetics

Binding kinetics were performed on the Carterra LSA^XT^ platform using the SAHC30M sensor chip (Carterra 4294) at 25 °C. First, the chip was conditioned using 1 min injections of 20 mM NaOH followed by 10 mM glycine (pH 2.5). Filtered and degassed 1X HBS-T (10 mM HEPES pH 7.4, 150 mM NaCl, 0.05% Tween-20; Carterra 3630) supplemented with 0.5 mg/ml of BSA (Sigma, A7030) was used as both running and sample diluent buffer (unless noted otherwise), and 1X HBS-T without BSA was used as running buffer for the multichannel side. Biotinylated Nbs were prepared in HBS-T–BSA supplemented with 150 mM NaCl (300 mM final [NaCl]) at eight concentrations (twofold dilution series spanning 100–0.781 ng/ml) and captured for 5 min on one of four prints. Fresh Nbs were captured for each CDTb titration series. CDTb constructs were prepared at six concentrations (fourfold dilutions) ranging from 300 nM to 0.3 nM using running buffer and injected over the chip surface from lowest to highest concentration without regeneration between points. Data collection times for baseline, association, and dissociation were 1 min, 5 min, and 10 min, respectively. The surface was regenerated between capture-titration sets using 3 × 30 s injection of 0.85% H_3_PO_4,_ followed by 3 × 30 s injection of 20 mM NaOH and 1 M NaCl to remove the bound antigen. Only freshly captured ligand surfaces were used for kinetic data analysis. Results were analyzed using Kinetics Software (Carterra). Data were double-referenced, serially Y-aligned, and fit to a one-to-one binding kinetics model. Data from three regions of interest with responses in 15 to 40 RU range were averaged to obtain mean on- and off-rate constants, and *K*_*D*_ values are shown in Table 1, Table 2, Table 3, Table 4. Figure 2, *A*–*D* shows representative sensorgrams for each combination of Nb-CDTb construct. Highest analyte concentrations showed non–one-to-one binding behavior in several datasets and were omitted.

### SPR epitope binning

Competitive binning assay was performed using Classical Binning wizard on Carterra LSA^XT^ platform (47) under the same conditions as described above for binding kinetics, with the following exceptions. Biotinylated Nbs were captured at 12 concentrations ranging from 5 μg/ml down to 0.002 μg/ml (twofold dilution series). Full-length CDTb_D1–D4_ was injected at 20 nM over the Nb array for 5 min, followed immediately by 5 min injection of 5 μg/ml of each nonbiotinylated Nb or buffer control. Nb array was regenerated after each cycle using 3 × 30 s injection of 0.85% H_3_PO_4_ followed by 3 × 30 s injection of 20 mM NaOH and 1 M NaCl. The results were analyzed using Epitope Software (Carterra). Data were referenced and Y-aligned; cutoffs were applied as described in the *Results* section. Only the highest-density ligand surfaces (capture concentrations of 5 μg/ml) were analyzed.

### Anti-CDTb ELISA

Ninety-six–well ELISA plates (Thermo Fisher Nunc MaxiSorp, 439454) were coated overnight at 4 °C on an orbital shaker with 200 ng/100 μl/well solutions of indicated capture Nbs diluted in PBS. The next day, the plates were washed four times (300 μl/well) with the wash buffer (PBS supplemented with 0.05% Tween-20 [PBS-T] and 0.08% NaN_3_) and incubated with blocking buffer (PBS-T + 2% BSA) for 1 h at room temperature with shaking, followed by four washes with wash buffer. For the toxin standard curves, CDTb (75 kDa-activated monomer) was diluted in a twofold dilution series in blocking buffer, added to the plate, and incubated for 1 h at room temperature with shaking. The plates were then washed four times with wash buffer. Next, 50 ng/100 μl/well solutions of indicated biotinylated detection Nbs diluted in PBS were added to the plates. After 1 h incubation at room temperature with shaking, the plates were washed four times with wash buffer. HRP-conjugated streptavidin (Jackson Immunoresearch, 016-030-084) was diluted 1:50,000 in blocking buffer, added to the plates (100 μl/well), and incubated for 1 h at room temperature with shaking and protection from light. After plates were washed five times with wash buffer, 75 μl/well of 1 step of UltraTMB ELISA substrate solution (Thermo Fisher, 34029, equilibrated to room temperature) was added to each well. Finally, reactions were quenched with 2 M H_2_SO_4_ in ∼2 min, and absorbance was measured at 450 nm in a Cytation plate reader (Biotek). The duplicate readings for each standard and sample were averaged, and the average zero standard absorbance was subtracted. Standard curves were constructed by interpolating the data using a sigmoidal four-parameter logistic curve fit (GraphPad Prism).

For CDTb standard curves in uninfected cecal and fecal backgrounds, cecal and fecal materials from PBS-treated uninfected mice were collected and flash frozen. After cecal material was resuspended in 1.5 ml of PBS and two to three fecal pellets were resuspended in 1 ml of PBS, the samples were macerated and incubated on ice for 30 min. The slurries were then diluted to 10 mg/ml in blocking buffer, which served as a diluent for twofold dilution series of CDTb. Toxin dilutions prepared in each background were then added to the ELISA plate.

### Quantification of CDTb in cecal material from infected mice

To obtain cecal material samples from infected mice, we used the cefoperazone model of mouse *C. difficile* infection (48). *C. difficile* growth conditions, spore preparation, generation of R20291 Δ*cdtB* mutant, and mouse infection model used in this study were recently described in detail (39). Briefly, 2 ml of overnight R20291 and R20291 Δ*cdtB* cultures were inoculated into 40 ml of Clospore medium (49), which was then grown for 5 days anaerobically. The spores were harvested, heat treated, and enumerated for the infection. Wildtype (JAX stock #664) C57BL/6J female mice were purchased between 8 and 10 weeks of age from Jackson Laboratories. Mice were given 0.5 mg/ml cefoperazone (Sigma, C4292) in sterile drinking water for 5 days. Antibiotic water was refreshed every other day to prevent antibiotic breakdown. After 5 days, mice were switched to regular water to recover for 2 days before inoculation. Mice were then orally gavaged with 10^3^ *C. difficile* spores. Mouse cecal material was harvested from humanely euthanized mice and snap frozen 1-, 2-, 3-, 4, and 7-day postinfection. The contents were resuspended in 1.5 ml of PBS, macerated, and incubated on ice for 30 min. The slurries were diluted to 10 mg/ml in PBS-T + 2% BSA, serially diluted twofold in PBS-T + 2% BSA, and added to prepared ELISA plates. ELISAs were performed as described above. Recombinant CDTb standard curve in PBS-T–BSA was included on each plate. The duplicate readings for each standard and sample were averaged, and the average zero standard absorbance was subtracted. Standard curves and sample concentrations were interpolated using a sigmoidal four-parameter logistic curve fit (GraphPad Prism). The measured concentration of samples calculated from the standard curve was converted to ng/ml, multiplied by the respective ELISA dilution factor, divided by the concentration of cecal material (0.01 g/ml), and reported as ng/g or pg/mg of cecal material. Mouse infection using both R20291 and R20291 Δ*cdtB* and subsequent sample harvest were independently performed two times.

### AlphaFold modeling

AlphaFold models were generated using AlphaFold3 (32). For CDTb, sequence entry consisted of 210 -to 876-residue range. For the Nbs, sequence entries started at “QVQ” residues and ended at “TVSSGS” residues (Table S2) and did not include any tags. All five models generated by AlphaFold for each complex are similar to each other and are overlaid in Fig. S8 (the only notable difference is the angle of positioning of D4 relative to the rest of the toxin because of its extended conformation and flexibility). Visualization and model mapping into the CDTb heptamer (Protein Data Bank ID: 6O2N) were performed in ChimeraX (50). Predicted aligned error matrix was visualized and extracted from PAE Viewer (51).

### Crosslinking MS

Each CDTb–Nb complex was incubated on ice for 30 min at a 1:1 M ratio in 1x PBS or 20 mM Hepes, pH 7.5. The chemical crosslinker, BS3 (bis(sulfosuccinimidyl)suberate; Thermo Scientific, A39266) was dissolved in 20 mM Hepes, pH 7.5 and added to each complex at 50 to 100X molar excess (final crosslinker concentration at 0.2–0.4 mM) of the antigen and Nb concentration. The reaction was incubated at room temperature for 1 h and quenched with a final concentration of 50 mM Tris, pH 8. The quenching reaction was incubated at room temperature for at least 15 min. Samples were separated *via* SDS-PAGE using an Invitrogen (Thermo Fisher Scientific) NuPAGE 4% to 12% Bis–Tris Gel. NuPAGE LDS Sample Buffer and DTT were added to each sample, and samples were incubated at 95 °C for 10 min before loading onto the gel. NuPAGE Mops running buffer was used. Gel was stained overnight with GelCode Blue Safe Protein (Thermo Scientific, 1860957).

Excised gel bands were diced into 1 mm^3^ cubes and in-gel digested using methods previously described by Tateishi *et al.* (52). Gel pieces were equilibrated in 100 mM NH_4_HCO_3_ (pH 8.0). Proteins were reduced in-gel with 4.5 mM DTT in 100 mM NH_4_HCO_3_ at 55 °C for 20 min and alkylated with 10 mM iodoacetamide in 100 mM NH_4_HCO_3_ at room temperature in the dark for 20 min. Gel pieces were destained with 50% CH_3_CN in 50 mM NH_4_HCO_3_, dehydrated by addition of 100% CH_3_CN, and dried in a speed vac concentrator. Proteins were digested with 10 ng/μl trypsin (Promega) in 25 mM NH_4_HCO_3_ at 37 °C overnight. Peptides were recovered by two extractions with 60% CH_3_CN/0.1% CF_3_CO_2_H. Extracts were dried, and peptides were reconstituted in 0.2% HCO_2_H for analysis by LC–MS/MS. A fused silica capillary column (360 μm O.D. × 100 μm I.D.) was packed with 20 cm of C18 reverse phase material (Jupiter, 3 μm beads; Phenomenex) directly into a laser-pulled emitter tip. Peptides were loaded onto the column using a Dionex Ultimate 3000 nanoLC and autosampler. Peptides were gradient eluted at a flow rate of 350 nl/min using a 90-min gradient. Mobile phase solvents consisted of 0.1% HCO_2_H, 99.9% H_2_O (solvent A), and 0.1% HCO_2_H and 99.9% CH_3_CN (solvent B). The gradient was as follows: 2% to 45% B in 70 min, 45% to 95% B in 7 min, 95% B for 2 min, 95 to 2% B in 1 min, and 2% B for 10 min. Peptides were analyzed on an Orbitrap Exploris 240 mass spectrometer (Thermo Scientific), equipped with a nanoelectrospray ionization source, using a top 20 data-dependent method. MS1 spectra were acquired at a resolution of 60,000 with an automatic gain control target of 3e6, followed by 20 MS/MS scans at a resolution of 15,000 with an automatic gain control target of 1e5 and an intensity threshold of 1e4. Precursors with charge 3 to 6 and undetermined charge states were fragmented using high-energy collisional dissociation at 30 normalized collision energy. Dynamic exclusion was enabled with an exclusion duration of 10 s.

Raw files were analyzed by the pLink2 (version 3.0.16) software for crosslinked peptides. The protein database consisted of the sequences of CDTb and each Nb (G2 and A9). The crosslinker was set to BS3, and the enzyme was set to trypsin. The following parameters were used: precursor mass tolerance, ±10 ppm; fragment mass tolerance, ±10 ppm; peptide length, 5 to 50; peptide mass, 600 to 6000, maximum number of missed cleavages; deamidation [N], oxidation [M], and carbidomethylation [C] were selected as variable modifications. Each reported crosslinked peptide was manually inspected and confirmed.

Visualization and crosslink mapping into AlphaFold models were performed in ChimeraX (50).

### *In vitro* oligomerization assay and size-exclusion chromatography

Full-length CDTb monomer was concentrated to 12.5 mg/ml. For each reaction, 1.5 mg of CDTb was mixed with nonbiotinylated Nb at a 5X molar excess and incubated for 30 min at room temperature. Then, bovine trypsin (Sigma, T1426) at a 1:5 (trypsin:toxin) ratio (w/w) was added to the complex, and the mixture was incubated with agitation at 37 °C for 45 min, with a final CDTb concentration being 7.9 mg/ml. Volume of CDTb and each Nb or buffer for mock treatment was kept the same across all reactions. PMSF was added at a 1 mM final concentration to quench the reaction, and the sample was applied to an S200 Increase 10/300 GL column in 20 mM Hepes (pH 8.0), 100 mM NaCl buffer to resolve double heptamer and activated CDTb monomer. Area under the curve of the double heptamer peak was calculated by the UNICORN (Cytiva) software. Fractions of interest were mixed with 4X SDS buffer, boiled at 100 °C for 5 min, and the same volume across all samples was loaded into 4% to 20% precast polyacrylamide gels (Bio-Rad MiniProtean TGX stain-free gels, 4568094). Gels were imaged on a Bio-Rad ChemiDoc MP Imaging System, and brightness adjustments were applied equally to all gels.

### *In vitro* flow cytometry cell binding assay

Vero cells (American Type Culture Collection, CCL-81) were maintained and cultured in 10 cm plates in DMEM + 10% heat-inactivated FBS at 37 °C with 5% CO_2_. On the day of the assay, CDTb-Alexa647 was preincubated with the Nbs for 1 h at room temperature in a 96-well plate. In the meantime, cells were washed three times with PBS before incubation for 30 min at 37 °C with Cellstripper dissociation solution (Corning, 25-056-CI). Cells were pipetted up and down, dissociation solution was diluted, with RPMI medium without FBS and phenol red, cells were harvested off each plate, and centrifuged (310*g* for 5 min). Then cells were resuspended in RPMI medium without FBS and phenol red and passed through a 70 Μm filter. Cells were then counted *via* a digital cell counter (Corning) and adjusted to 1.25 × 10^6^ cells/ml. 7-Aminoactinomycin D (7-AAD) viability dye (Thermo, A1310) was added to the cells at 0.125 μg/million cells, and cells were distributed at 200,000 cells/200 μl/well in a 96-well plate containing preincubated toxin and Nbs (final concentrations: CDTb at 25 Nm and each Nb at 100 Nm). In addition, each experiment contained cells treated for compensation controls (7-AAD^-^, CDTb-Alexa 647^-^ cells; 7-AAD^+^, CDTb-Alexa 647^-^ cells; 7-AAD^-^, CDTb-Alexa 647^+^ cells). Ninety-six–well plate was incubated at 37 °C for 90 min. Cells were harvested and washed twice with cold PBS by centrifuging the plate (400*g* for 5 min). Cells were transferred into flow tubes, and data were immediately collected on a 3-laser BD Fortessa cytometer. The gating strategy is shown in Fig. S11. Analyzed data are presented using FlowJo 10.10.0 (FlowJo, LLC).

## Data availability

The data described are contained within the article. AlphaFold3 models were deposited to ModelArchive (53). The models are available in ModelArchive with the accession codes ma-etzgn (CDTb + A9), ma-8rcuw (CDTb + C6), ma-9xfr0 (CDTb + G2), ma-vzani (CDTb + H12). Sequences of Nbs are listed in the Tables S1 and S2. The MS proteomics data have been deposited to the ProteomeXchange Consortium *via* the PRIDE (54) partner repository with the dataset identifier PXD070923.

## Supporting information

This article contains supporting information.

## Conflict of interest

B. W. S. and B. E. W. are founders and principals at Turkey Creek Biotechnology LLC (Waverly, TN). Turkey Creek Biotechnology performed the alpaca immunizations and blood draws but was not involved in subsequent work. M .M. is an employee at Carterra, Inc. The authors declare that they have no conflicts of interest with the contents of this article.
