# Supplementary material for: Nanobodies against Clostridioides difficile CDTb provide a toolkit for potent toxin neutralization and highly sensitive quantitation
Source: J Biol Chem. 2025 Dec 22;302(2):111082. doi: 10.1016/j.jbc.2025.111082 (PMC12828747; doi:10.1016/j.jbc.2025.111082)
Supplement: Supporting information [file mmc1.pdf]

Supporting Information

Nanobodies against *Clostridioides difficile* CDTb provide a toolkit for potent toxin neutralization and highly sensitive quantitation

Kateryna Nabukhotna, Heather K. Kroh, David M. Anderson, Rubén Cano Rodríguez, John A. Shupe, Maria McGresham, Carla VT. O'Neale, Rebecca A. Shrem, Brian E. Wadzinski, Kevin L. Schey, Benjamin W. Spiller, D. Borden Lacy

Table of Contents

Supplementary Figure 1 .... S-2
Supplementary Figure 2 .... S-7
Supplementary Figure 3 .... S-8
Supplementary Figure 4 .... S-9
Supplementary Figure 5 .... S-10
Supplementary Figure 6 .... S-11
Supplementary Figure 7 .... S-12
Supplementary Figure 8 .... S-14
Supplementary Figure 9 .... S-16
Supplementary Figure 10 .... S-20
Supplementary Figure 11 .... S-23
Supplementary Figure 12 .... S-25
Supplementary Table 1 .... S-26
Supplementary Table 2 .... S-29
Supplementary Table 3 .... S-30
Supplementary Table 4 .... S-30

Supplementary Figure 1

A

```
             1 ........10........20........30........40........50........60
C1D1         1 QVQLQESGGGLVQPGRSLRLSCEASDFSLNTFGA--GWFRQVPGKEREGVSCISPS--NS
C1H7         1 QVQLQESGGGLVQPGRSLRLSCEASDFSLNTFGA--GWFRQVPGKEREGVSCISPS--NS
C1H8         1 QVQLQESGGGLVQPGRSLRLSCEASDFSLNTFGA--GWFRQVPGKEREGVSCISPS--NS
C1A5         1 QVQLQESGGGLVQPGGSLRLSCAASGFTFVDYAM--GWIRQATGKNNEGVSCVSGS--GR
C1D10        1 QVQLQESGGGLVQPGGSLRLSCAVSGLAXDYYAI--GWFRQAPGKEREEVSCLNGS--DG
C1B2         1 QVQLQESGGGLVQPGGSLRLSCADSGTTFIYYAM--GWFRQAPGKEREVVACINGN--GD
C1B4         1 QVQLQESGGGLVQPGGSLRLSCTDAGTTAIYYAI--GWFRRAPGKEREGIACINMN--GD
C1D3         1 QVQLQESGGGLVQPGESLRLSCTASGFTLDRYHI--GWFRQAPGKEREGVACISSN--LG
C1D12        1 QVQLQESGGGLVQPGESLRLSCTASGFTLDRYHI--GWFRQAPGKEREGVACISSN--LG
C1H2         1 QVQLQESGGGLVQPGGSLRLSCAASGFSLDYHAI--GWFRQAPGKEREGVSCITS---TG
C1F9         1 QVQLQESGGGLVQPGGSLRLSCTASGSTSDLYTT--GWFRQAPGKEREGVSCISFS--SG
C1F10        1 QVQLQESGGGSVQPGGSLRLSCAPTGSSLDYYAI--GWFRQAPGKEREVVATITNT--GG
C1B5         1 QVQLQESGGGLVQPGGSLTLSCAASGSIGSGSVM--SWYRQAPGKERELVAQES-G--GR
C1G1         1 QVQLQESGGGLVQPGGSLTLSCAASGSIGSGSVM--SWYRQAPGKERELVAQES-G--GR
C1H6         1 QVQLQESGGGLVQPGGSLTLSCAASGSIGSGSVM--SWYRQAPGKERELVAQES-G--GR
C1E5         1 QVQLQESGGGLAQPGGSLRLSCEVSGFDFSSSDM--SWHRQTPGNERELVAAIASR--SG
C1C4         1 QVQLQESGGGLVQPGGSLRLSCAASGYTINSLTV--AWFRQAPGKEREFIAASGAP--DN
C1C10        1 QVQLQESGGGLVQSGGSLRLSCAASGSPITTLTV--AWFRQAPGKEREFIAASGSP--DN
C1D9         1 QVQLQESGGGLAQTGNSLRLSCAASGGTIGTLTV--AWFRQAPGKEREFVAATGVP--DN
C1G6         1 QVQLQESGGGLVQPGGSLRLSCASSGFAFSKYSM--TWVHQAPGKGLEWVSQITGS--AD
C1H11        1 QVQLQESGGGLVQPGGSLRLSCAASGFTFGSYVV--QWYRQPPGKERELVAQIRVT--DN
C1A8         1 QVQLQESGGGLVQPGGSLRLSCTTSGFTFSTYNM--KWYRQAQGKERELVATISNA--NN
C1D11        1 QVQLQESGGGLIQPGGSLRLSCVASGFTFSTATI--KWYREAPEKERELVALIGNT--GG
C1F7         1 QVQLQESGGGLVQTGDSLRLSCEVSGATFGDYRLSTAWFRQAPGKDREFVAIIMRL---G
C1A12        1 QVQLQESGGGSVEIGGSVNLSCAASGGTFRRYTM--AWFRQVPGKSREFVAARQRD---G
C1A6         1 QVQLQESGGGLVQPGGSLRLSCVAAGTDFKHNLM--GWYRQAPGKQRELVASVLGSGGGG
C1A9         1 QVQLQESGGGLVQPGGSLRLSCTSSTNMLEFTAV--AWWRQAPGKQRDLLAVITRA---G
C1B7         1 QVQLQESGGGLVQPGGSLRLSCTSSTNMLEFTAV--AWWRQAPGKQRDLLAVITRA---G
C1E11        1 QVQLQESGGAMVQPGGSLRLSCAASGSGSNSSAM--AWYRQAPGKQRELIAVITRA---N
C1G2         1 QVQLQESGGGLVQPGGSLTLSCKASGIVFSTHTL--AWYRQAPGKHRNVLAVITSA---G
C1B9         1 QVQLQESGGGLVQPGGSLRLSCVASGFTFSRYDM--SWHRQAPGKERELVASTRTM-TGT
C1E4         1 QVQLQESGGGLVQPGGSLRLSCVVSGSTLDHYTI--GWFRQAPGKEREGVAWISMN--YG
C1F11        1 QVQLQESGGGLVQPGGSLRLSCAASADISVYTTL--GWYRQAPGKERELVAQTTSR---G
C1H12        1 QVQLQESGGGLVQPGGSLRLSCAASGDIFSFYTM--GWYRQAPGKQRELVATITSP---G
C1C1         1 QVQLQESGGGLVQPGGSLRLSCVASGGIFSIYAM--SWYRQAPGKQRELVATIATY---G
C1D6         1 QVQLQESGGGLVQPGGSLRLSCTASGGIFSIYAM--NWYRQAPGKQRELVASISTL---S
C1D8         1 QVQLQESGGGLVQAGDSLRLSCAASEGTFETYGM--GWFRQAPGREREFVASVNWL--GG
C1C6         1 QVQLQESGGGLVEPGGSLRLSCVASGIVVASSTM--SWYRQAPGKQRELVAQI-FS--GG
C1F5         1 QVQLQESGGGLVEPGGSLRLSCVASGIVVASSTM--SWYRQAPGKQRELVAQI-FS--GG
C1G8         1 QVQLQESGGGLVQAGDSLRLSCAASGRTFSGYAM--GWFRQAPGKEREVVAAISWI--AG
C1H10        1 QVQLQESGGGLVQAGDSLRLSCAASGRT----YAR--AWFRESPGKEREFVAAIGIG--GI
C1D7         1 QVQLQESGGGLVQAGDSLRLSCANSGRTFSSYGL--AWFREAPGKEREFVAALRW----G
C1E6         1 QVQLQESGGGLVQAGDSLRLSCANSGRTFSSYGL--AWFREAPGKEREFVAALRW----G
C1E7         1 QVQLQESGGGLVQAGDSLRLSCANSGRTFSSYGL--AWFREAPGKEREFVAALRW----G
C1B11        1 QVQLQESGGGLVQAGDSLRLSCAASERTFRTYAM--GWFREAPGKEREFVAALRWF--LG
C1A3         1 QVQLQESGGGLVQAGDSLRLSCAASGRSFSTYAM--GWFREAPGKEREFVATFRWF--SG
C1A11        1 QVQLQESGGGLVQAGDSLRLSCAASGRTFSTYAM--GWFRETPGKEREF--TFRWF--SG
C1B12        1 QVQLQESGGGLVQAGDSLRLSCAASGRTFSTYAM--GWFREAPGKEREFVATFRWF--SG
C1E9         1 QVQLQESGGGLVLAGDSLRLSCAASGRTFSTYAM--GWFREAPGKEREFVATIRWF--LG
C1B1         1 QVQLQESGGGLVQAGDSLTLSCAASGRTFSTYAM--GWFREAPGKEREFVATVRWF--LG
C1G4         1 QVQLQESGGGLVQAGDSLTLSCAASGRTFSTYAM--GWFREAPGKEREFVATVRWF--LG
C1E10        1 QVQLQESGGGLVQTGDSLTLSCAASGRTFSTYAM--GWFREAPGKEREFVATVRWF--LG
```

```
C1A7     1  QVQLQESGGGLVQAGGSLRLSCANSGRTLNSFAM--GWFRQAAGKEREFVAAISRI--AG
C1F8     1  QVQLQESGGGLVQAGGSLRLSCANSGRTLNSFAM--GWFRQAAGKEREFVAAISRI--AG
C1C3     1  QVQLQESGGGLVQAGGSLRLSCAASGRTFSSYAA--GWFRQAPGNEREFVSAINKL--GT
C1A4     1  QVQLQESGGGLVQAGDSLRLSCAFSGDTFSSYTM--GWFRQAPGKEREFVAAFSGI--GT
C1C5     1  QVQLQESGGGLVQAGDSLRLSCAFSGDTFSSYTM--GWFRQAPGKEREFVAAFSAI--GT
C1H9     1  QVQLQESGGGSVQAGGSLKLSCAASAGTFSNYAL--GWFRQAPGKEREFVAHISWI--GG
C1B3     1  QVQLQESGGGSVQAGGSLKLSCAASAGTFSNYAL--GWFRQAPGKEREFVAHISWI--GG
C1G10    1  QVQLQESGGGLVQAGGSLXLSCAASGGTFSNYAL--GWFRQAPGKERELVAHISWI--GG
C1G5     1  QVQLQESGGGLVQAGDSLRLSCAASEHTFSNYAL--AWFRQVPGQEREFVAHISWI--GG
C1B6     1  QVQLQESGGGLVQSGGSLRLSCAASDSTFSNYAL--GWFRQAPGKEREFVAHIRWI--GG
C1C11    1  QVQLQESGGGTVQAGGSLRISCAASGRTASNYAM--GWFRQAPGKEREFVAHIRWL—GG

        61  ........70........80........90.......100.......110.......120
C1D1    57  56STYYADSVKGRFTISRDNAKNTIYLQMNSLKPEDTGVYFCAARPITSAQALCTLLVS
C1H7    57  56STYYADSVKGRFTISRDNAKNTIYLQMNSLKPEDTGVYFCAARPITSAQALCTLLVS
C1H8    57  56STYYADSVKGRFTISRDNAKNTIYLQMNSLKPEDTGVYFCAARPITSAQALCTLLVS
C1A5    57  56MTDYADSVKGRFTISRDNAKNMVYLQMNNLKPEDTGVYYCVASW-DDGYGXSC----
C1D10   57  56XTYYGDSVKGRFTISRDNAKNTVYLQLNRLXPEDXGVYYCAAXRGGWGCL-TDSFSX
C1B2    57  56NPYYADSVRGRFTISRDDAKNTVYLQMNGLKPEDAAVYYXAAANDFDG---RCYXXS
C1B4    57  56GTNYADSVKGRFTISRDDAKNTVYLQMNSLKSEDAAVYYCAAANDFDG---RCNLRS
C1D3    57  56TTNYADSVKGRFTISRDNAKNTVTLQMNSLTPEDTGVYYCAGQPR-ANMFAYSFCAP
C1D12   57  56TTNYADSVKGRFTISRDNAKNTVTLQMNSLTPEDTGVYYCAGQPR-ANMFAYSFCAP
C1H2    56  55VTNYADSAKGRFTISRDNLRNTMYLQMNSLKPEDTAVYYCAAAPP---GQLYRLCEP
C1F9    57  56STDYADSVKGRFTISRDDAKNTVYLQMNNLKPEDTAVYYCGLIAWLEGDGSSCLKRA
C1F10   57  56LTSYADSVKGRFTISRDNAKNTVYLQMNSLKFEDTAVYYCELRN-------------
C1B5    56  55LRSYAKSVMGRFTISRDNSNNTVTLQMNNLKTDDTAIYYCHLV-------------Q
C1G1    56  55LRSYAKSVMGRFTISRDNSNNTVTLQMNNLKTDDTAIYYCHLV-------------Q
C1H6    56  55LRSYAKSVMGRFTISRDNSNNTVTLQMNNLKTDDTAIYYCHLV-------------Q
C1E5    57  56LISYKDSVKGRFTVSRDNAKNTVYLQMSSLKPEDTGTYYCNVGFR-----------V
C1C4    57  56LTGYQNSLKGRFTISRDNAKNTLYLQMTSLKPEDTAVYYCAARPGPYS------SQT
C1C10   57  56LTGYTPSLKGRFTISRDNAKNTLYLQMTSLNPEDTAVYYCAARPGPYS------SQT
C1D9    57  56LTGYTPSLKGRFTISRDNAKNALYLQMTTLKPEDTAVYYCAARPGPYS------SRT
C1G6    57  56NIYYADSVKGRFIISRDNAKNTLYLQMNSLKPEDTGVYYCAIDI------------R
C1H11   57  56LISYKNFAKGRFTISTDNTKNTVYLQMNSLTPEDTAVYYCRYQ--------------
C1A8    57  56LISYAPSVRGRFTISRDNAKNTLYLQMNSLKPEDTAEYYCNAQ-------------N
C1D11   57  56LTSYAPSVKGRFTVSRDNAKSTVWLQMNTLKPEDTAMYFCNAQISGA--------PY
C1F7    58  57DTYYSDSMKGRFTISRDDTGNTLYLQMNGLNPEDTAVYFCAAGEQYYCSP-DRCKNT
C1A12   56  55LILYEPSVKGRFAISRDNAENTLTLQMNNLRVEDTAIYYCAARDI----M-AFSRDP
C1A6    59  58LITYGDPVKGRFTISRDSAQNSVDLQMNNLHPEDTAIYYCHAKKM------------
C1A9    56  55VPNYADS-KGRFAISRDNAKNTVDLLINTLEPDDTAVYTCNARTS------------
C1B7    56  55VPNYADS-KGRFAISRDNAKNTVDLLINTLEPDDTAVYTCNARTS------------
C1E11   56  55VPNYVPSMKDRFTISRDNAKNTAYLQIDSLKAEDTAVYYCNARTTTG----------
C1G2    56  55IPNYDTDLKGRFTISRDNAKNTVYLQMNSLEPEDTAVYYCNARGPHPT------VTG
C1B9    58  57GVNYADSVKGRFTMSIDGAKNTVYLQMNSLKPEDTAVYYCHAERK-YA------YYD
C1E4    57  56RTNYADSAKGRFTISRNNAENTVYLQMTSLEPMDTAVYYCAAEDSTYG---SAWPND
C1F11   56  55DTDYADSVKGRFTISRDNAKNMLYLXMDSLKPEDTAVYYCNAKYT---------VNW-
C1H12   56  55TTNYADSVKGRFTISRDNAKNMVYLQMTSLKPEDTAVYYCNADRI---------LTTD
C1C1    56  55TADYEDAVKGRFTISRDNAKNTVYLQMNSLKPEDTAVYYCYADPE---------MGVA
C1D6    56  55TTDYADSVKGRFTISRDNAKNAVYLQMNSLKPEDTAVYYCNADPE---------MGVA
C1D8    57  56NVVYAPSVKGRFTISRDNTKNMLYLQMNSLNPMDTAVYYCAASQSQYQ-V-GFTTRS
C1C6    56  55NTNYRDSVRGRFTISKDNAKNTAYLQMDNLEPDDTAVYYCYAR--------------
C1F5    56  55NTNYRDSVRGRFTISKDNAKNTAYLQMDNLEPDDTAVYYCYAR--------------
C1G8    57  56STHYADFAKGRFTISRDKAKNTLFLQMNSLKPEDTAVYYCAAAGKENP-Y-PLIWEE
C1H10   54  53TTYYANSVKGRFTISRDNAKNTLYLQMDSLKPEDTAVYYCAATGG-SIGY-FIAENE
C1D7    55  54NPYYADSVKGRFTISRDNAKTTLYLQMNSLKPEDTGVYYCAATEG-SSGY-FIAENE
C1E6    55  54NPYYADSVKGRFTISRDNAKTTLYLQMNSLKPEDTGVYYCAATEG-SSGY-FIAENE
C1E7    55  54NPYYADSVKGRFTISRDNAKTTLYLQMNSLKPEDTGVYYCAATEG-SSGY-FIAENE
```

```
C1B11    57   56NTYYADSVKGRFTISKDDAKNTLYLLMNSLQPDDTAVYYCAATHGTTSGY-FIPDNE
C1A3     57   56ITYYAESVKGRFTISRDNAKNTVYLQMNSLKPEDTAVYYCAATRGTTSGY-FIPENE
C1A11    55   54RTYYADSVKGRFTISRDNAKNTVYLQMNSLKPEDTAMYYCAATAGTTSGY-FIPEND
C1B12    57   56RTYYADSVKGRFTISRDSAKNTVYLQMNSLKPEDMAMYYCAATAGTTSGY-FIPEND
C1E9     57   56NTYYADSVKGRFTISKDNAKNTIYLQMNSLKPEDTAMYYCAATAGTTSGY-FIPENE
C1B1     57   56NTYYADSVKGRFSISKDNAKNTVYLQMNSLKPEDTAVYYCAATKGTTSGY-FIPEND
C1G4     57   56NTYYADSVKGRFSISKDNAKNTVYLQMNSLKPEDTAVYYCAATKGTTSGY-FIPEND
C1E10    57   56NTYYADSVKGRFSISKDNAKNTVYLQMNSLKPEDTAVYYCAATKGTTSGY-FIPEND
C1A7     57   56-TSYADSVKGRFTISKDYAKNTLYLQMTSLKPEDTAVYYCAATAP-SGYV-I-LEMD
C1F8     57   56-TSYADSVKGRFTISKDYAKNTLYLQMTSLKPEDTAVYYCAATAP-SGYV-I-LEMD
C1C3     57   56STYYEDSVKGRFTISRDNAKNTLYLEMNSLKPEDTAVYYCAATAD-PSWY-TMPGNE
C1A4     57   56GTYYADYVKGRFTISRDNAKNTLYLQMNNLKPEDTAVYYCAATTG-GTYY-I-FELE
C1C5     57   56GTYYADYVKGRFTISRDNAKNTLYLQMNNLKPEDTAVYYCTATTG-GTYY-I-FELE
C1H9     57   56RTNYADSVKGRFTISRDNAKNTAYLQMDNLEPDDTAVYYCYA---------------
C1B3     57   56RTNYADSVKGRFTISRDNAKNTLYLQMNGLEIEDTAVYYCAACRG-GAYY-V-FSHA
C1G10    57   56PTNYADSVKGRFTISRDNAKNTLYLLMNSLEIEDTAVYYCAACHG-GAKY-L-FSHS
C1G5     57   56KTNYADSVKGRFTISRDNAKNTLYLQMNSLKPEDTAVYYCAACRG-GSYY-I-HSSE
C1B6     57   56STEYANSVKGRFTISRDNAKSTLYLQMNSLKPEDTAVYYCAAVRG-GSYY-I-ASSE
C1C11    57   56STEYADSVKGRFTISRDNAKNTLYLQMNGLKPEDTAVYYCAAVRG-GSYY-I-HDNE

              121 .......130.......140...
C1D1    114 DSF     116TSWGQGTQVTGS     128
C1H7    114 DSF     116TSWGQGTQVTGS     128
C1H8    114 DSF     116TSWGQGTQVTGS     128
C1A5    109 ITP     111EYRGQGTQVTGS     123
C1D10   113 AEY     115DYWGQXTQVTGS     127
C1B2    111 LWY     113XDWXQGTXVTGS     125
C1B4    111 PWY     113SDWGQGTQVTGS     125
C1D3    113 YEY     115DYWGQGTQVTVS     127
C1D12   113 YEY     115DYWGQGTQVTVS     127
C1H2    110 YEY     112DYWGQGTQVTGS     124
C1F9    114 WEY     116DYWGQGTQVTGS     128
C1F10   101 VGN     103TYWGQGTQVTGS     115
C1B5    100 FSG     102RYWGHGTQVTGS     114
C1G1    100 FSG     102RYWGHGTQVTGS     114
C1H6    100 FSG     102RYWGHGTQVTGS     114
C1E5    103 SSL     105ASWGQGTQVTVS     117
C1C4    108 RDY     110PYWGQGTQVTGS     122
C1C10   108 RHY     110PYWGQGTQVTGS     122
C1D9    108 TDY     110PYWGQGTQVTGS     122
C1G6    102 WDN     104AYWGQGTQVTGS     116
C1H11   100 ---     99DNWGQGTQVTGS      111
C1A8    101 FLS     103NYWGQGTQVTGS     115
C1D11   106 FGE     108NFWGQGTQVTGS     120
C1F7    114 NDY     116DYWGQGTPVTGS     128
C1A12   108 QNY     110DYWGQGTQVTGS     122
C1A6    104 -GT     105DYWGPGTQVTGS     117
C1A9    100 GNI     102NYWGQGTQVTGS     114
C1B7    100 GNI     102NYWGQGTQVTGS     114
C1E11   103 --V     103NYWGQGTQVTGS     115
C1G2    107 TTA     109NYWGQGTQVTGS     121
C1B9    108 PEY     110DYWGQGTQVTGS     122
C1E4    111 WXM     113DYWGPGTQVTGS     125
C1F11   104 YEN     106DYWGQGTQVTGS     118
C1H12   105 PQF     107KYWGQGTQVTGS     119
C1C1    105 GMK     107DYWGQGTQVTGS     119
C1D6    105 GMR     107EYWGQGTQVTGS     119
```

```
C1D8    112 ADF     114GSWGQGTQVTGS    126
C1C6     99 ---     98GFWGQGTQVTGS     110
C1F5     99 ---     98GFWGQGTQVTGS     110
C1G8    112 YEY     114DYWGQGTQVTGS    126
C1H10   109 AEY     111DYWGQGTQVTGS    123
C1D7    110 GEY     112DYWGQGTQVTGS    124
C1E6    110 GEY     112DYWGQGTQVTGS    124
C1E7    110 GEY     112DYWGQGTQVTGS    124
C1B11   113 GEY     115DYWGQGTQVTGS    127
C1A3    113 GEY     115DYWGQGTQVTGS    127
C1A11   111 GEY     113DYWGQGTQVTGS    125
C1B12   113 GEY     115DYWGQGTQVTGS    127
C1E9    113 GEY     115DYWGQGTQVTGS    127
C1B1    113 GEY     115DYWGQGTQVTGS    127
C1G4    113 GEY     115DYWGQGTQVTGS    127
C1E10   113 GEY     115DYWGQGTQVTGS    127
C1A7    110 TEY     112DYWGQGTQVTGS    124
C1F8    110 TEY     112DYWGQGTQVTGS    124
C1C3    112 GEY     114DYWGQGTQVTGS    126
C1A4    111 TEY     113DYWGQGTQVTGS    125
C1C5    111 TEY     113DYWGQGTQVTGS    125
C1H9     99 --R     99GFWGQGTQVTGS     111
C1B3    111 AEY     113DYWGQGTQVTGS    125
C1G10   111 AEY     113DFWGQGTHVTGS    125
C1G5    111 AEY     113DYWGQGTQVTGS    125
C1B6    111 AEY     113DYWGQGTQVTGS    125
C1C11   111 AEY     113DYWGQGTQVTGS    125
```

B

```
          1 ........10........20........30........40........50........60
C1H8      1 QVQLQESGGGLVQPGRSLRLSCEASDFSLNTFGAGWFRQVPGKEREGVSCISPSNSSTYY
C1A9      1 QVQLQESGGGLVQPGGSLRLSCTSSTNMLEFTAVAWWRQAPGKQRDLLAVITRA-GVPNY
C1G2      1 QVQLQESGGGLVQPGGSLTLSCKASGIVFSTHTLAWYRQAPGKHRNVLAVITSA-GIPNY
C1C6      1 QVQLQESGGGLVEPGGSLRLSCVASGIVVASSTMSWYRQAPGKQRELVAQIFSG-GNTNY
C1H12     1 QVQLQESGGGLVQPGGSLRLSCAASGDIFSFYTMGWYRQAPGKQRELVATITSP-GTTNY

         61 ........70........80........90.......100.......110.......120
C1H8     61  60ADSVKGRFTISRDNAKNTIYLQMNSLKPEDTGVYFCAARPITSAQALCTLLVSDSFT
C1A9     60  59ADS-KGRFAISRDNAKNTVDLLINTLEPDDTAVYTCNAR------------TSGNIN
C1G2     60  59DTDLKGRFTISRDNAKNTVYLQMNSLEPEDTAVYYCNARGPHP------TVTGTTAN
C1C6     60  59RDSVRGRFTISKDNAKNTAYLQMDNLEPDDTAVYYCYAR-----------------G
C1H12    60  59ADSVKGRFTISRDNAKNMVYLQMTSLKPEDTAVYYCNADRILT--------TDPQFK

        121 .......130.........
C1H8    118 SWG     120QGTQVTGS 128
C1A9    104 YWG     106QGTQVTGS 114
C1G2    111 YWG     113QGTQVTGS 121
C1C6    100 EWG     102QGTQVTGS 110
C1H12   109 YWG     111QGTQVTGS 119
```

Figure S1. Nanobody sequence alignment. Amino acid sequence alignments of (**A**) 63 clones and (**B**) 5 clones further characterized in the study. Boxes next to the nanobodies indicate domain specificity based on ELISA results shown in panel Figure 1C (blue D1-D3, red D3’ and green D4). Residues highlighted in black are identical residues, and residues highlighted in grey

are similar residues. Alignments of amino acid sequences were performed in CLUSTAL OMEGA.

Supplementary Figure 2

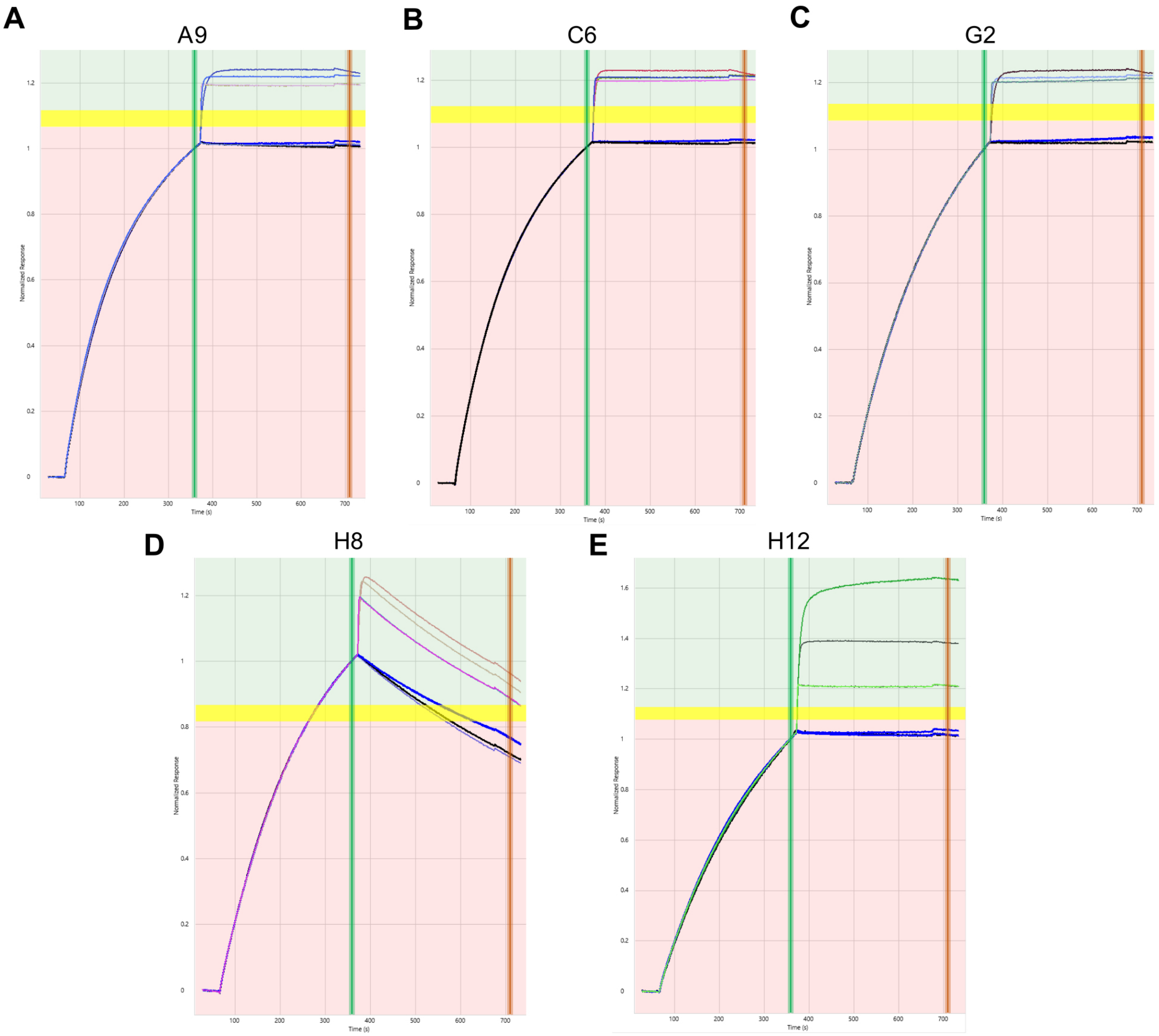

Figure S2. Representative normalized sensorgram overlay plots from epitope binning experiment. Representative normalized data for a single ROI of each captured ligand nanobody (labeled at the top). Data were referenced and globally Y-aligned. Each sensorgram was normalized to CDTb antigen binding (shown by the green vertical line). Black sensorgram identifies self-self interaction. Antigen only controls are shown in blue. Orange vertical bar indicating binding response report point was placed at the end of analyte nanobody injection. Competition threshold shown as a horizontal yellow line was set at 0.05-0.1. Any report points falling below the yellow bar indicate blocking; those falling above the bottom edge of the yellow bar are considered non-blocking.

Supplementary Figure 3

A

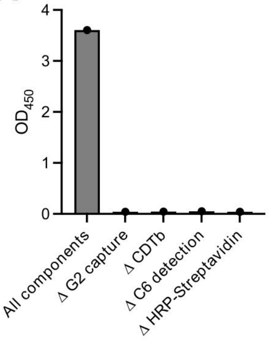

B

Nonlin fit<br>Table of results	A<br>Mock	B<br>Cecal material	C<br>Global (shared)
	Y	Y	Y
**Comparison of Fits**			
Null hypothesis			One curve for all data sets
Alternative hypothesis			Different curve for at least one data set
P value			0.1205
Conclusion (alpha = 0.05)			Do not reject null hypothesis
Preferred model			One curve for all data sets
F (DFn, DFd)			1.904 (4, 64)

Figure S3. Control experiments for ELISA development. (**A**) Control experiment demonstrating specificity of nanobody-based sandwich ELISA. The following setup was used: G2 (capture) → purified CDTb → C6 (detection) → HRP-Streptavidin → TMB. OD_{450} values demonstrating ELISA outcomes in the presence of all components or absence of G2 capture, purified CDTb, C6 detection, and HRP-Streptavidin during the reaction. The experiment was performed once. (**B**) Comparison of best-fit values of curve-fit parameters between mock and cecal material standard curves. Summary of extra sum-of-squares F test showing that one curve fits both data sets. This indicates that the difference in fit parameters (bottom, top, IC_{50}, and Hillslope) of two standard curves is not statistically significant (at $P < 0.05$), and thus the data sets do not different from each other. The analysis was performed in GraphPad Prism.

Supplementary Figure 4

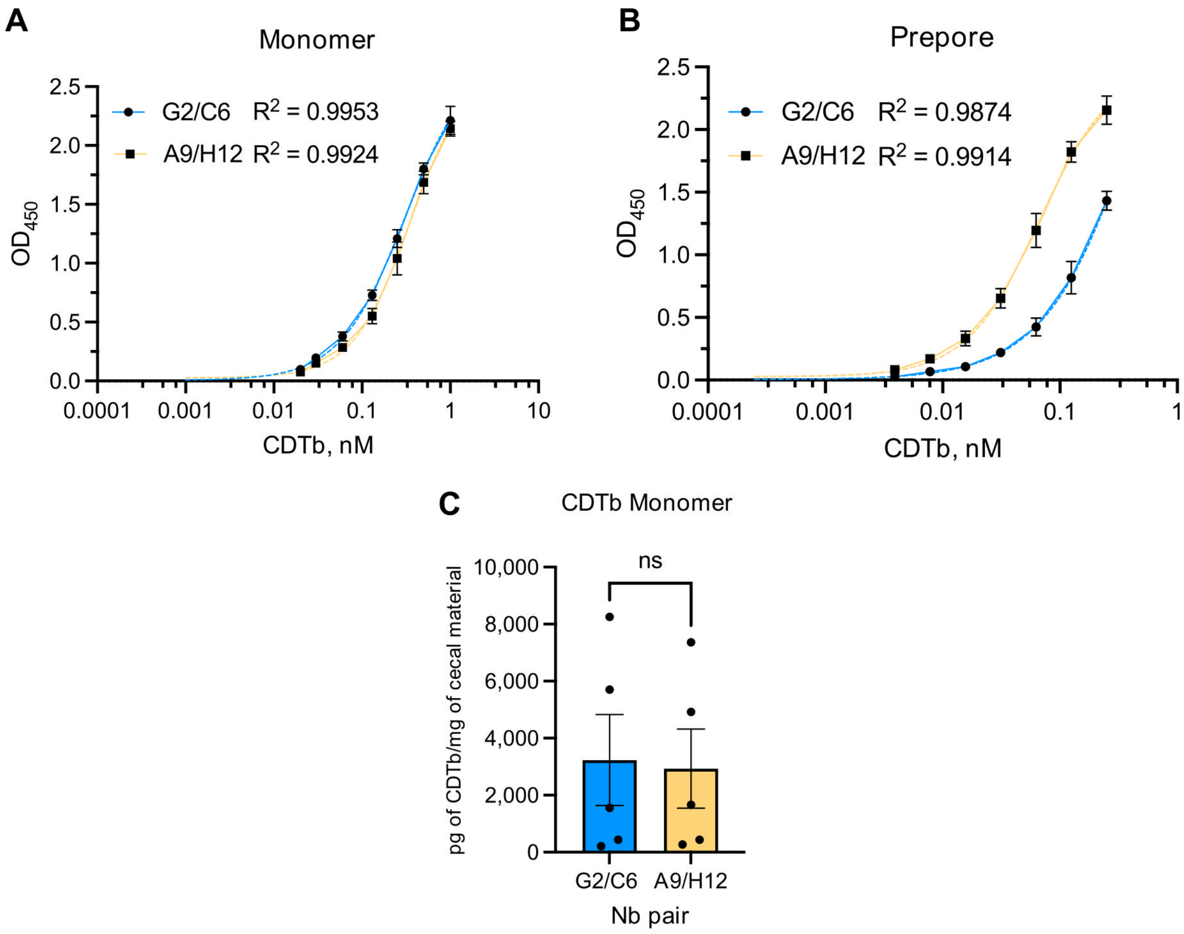

Figure S4. Control experiments addressing monomer vs heptamer recognition by Nb C6. (**A**) A9/H12 Nb pair recognizes monomeric CDTb similarly to G2/C6 pair. (**B**) A9/H12 Nb pair recognizes heptameric CDTb more efficiently than G2/C6 pair. We hypothesize that the background signal produced by G2/C6 Nb pair is due to residual contamination of monomeric CDTb in the heptameric sample or due to disassembly of some heptamers into monomers within the sample. Standard curves were constructed by interpolating the data using sigmoidal four parameter logistic (4-PL) curve-fit. Data points are connected by solid lines, and color-matched curve fits are shown in dashed lines. Each data point represents mean ± SD of three independent biological experiments (n = 3). Each biological experiment consisted of two technical replicates, and the average value of both technical replicates was used. (**C**) Most of CDTb in cecal material 4 days post R20291 infection is found in monomeric state given that G2/C6 pair's sensitivity is comparable to the A9/H12 pair. For the standard curve in this assay, monomeric CDTb was used. Bars represent mean ± SEM of the group; dots represent an individual mouse within the group. Two-tailed unpaired t-test was used to calculate statistical significance (ns = 0.8914). *In vivo* experiments were independently performed 2 times (with 2–3 animals per each group).

Supplementary Figure 5

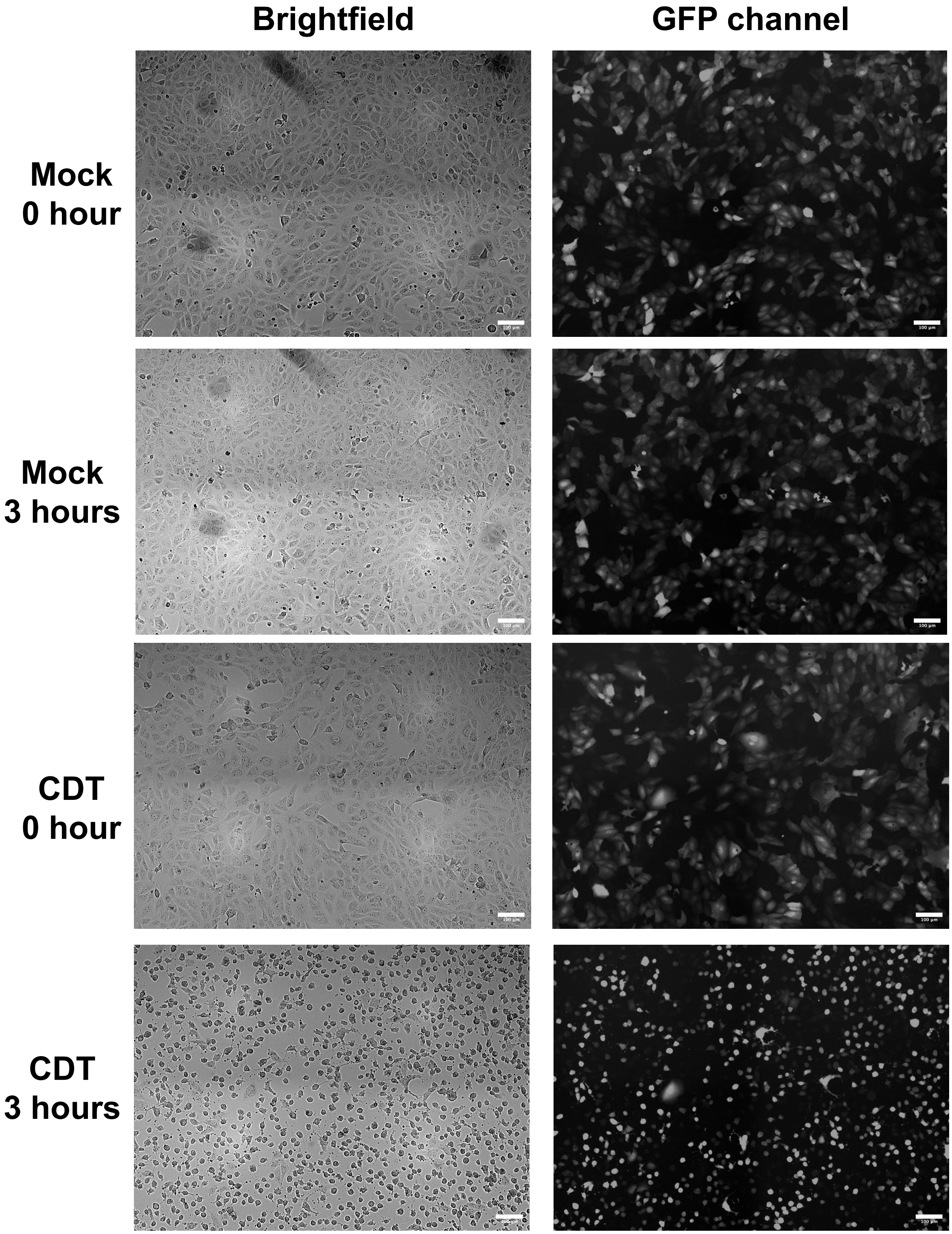

Figure S5. Representative images of Vero-GFP cells treated with CDT. Vero-GFP cells (left – brightfield, right – GFP channel) treated with vehicle or CDT at 3 hours post-intoxication. Scale bar: 100 μm.

Supplementary Figure 6

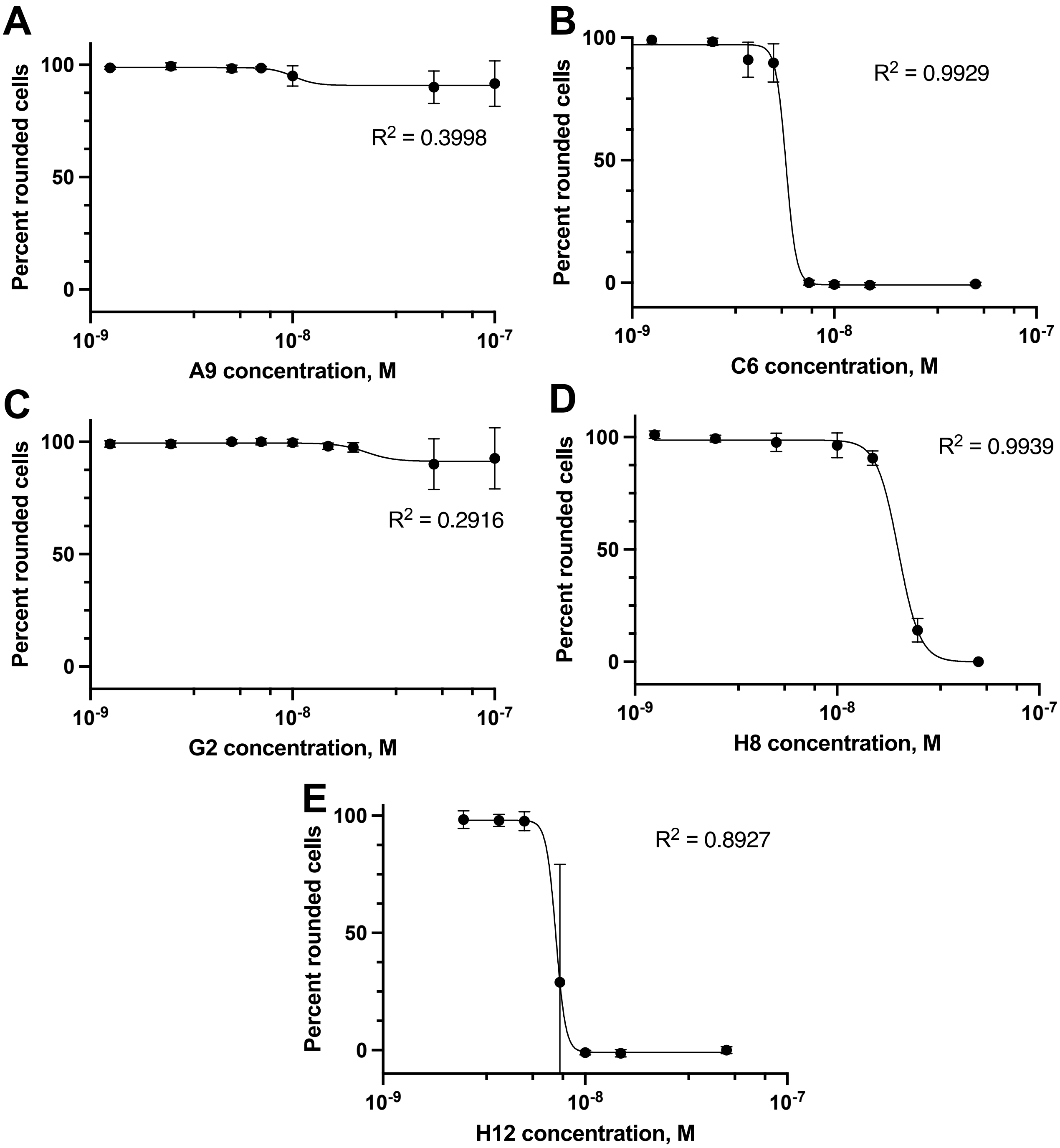

Figure S6. Neutralization potency of anti-CDTb nanobodies at 8 hours post-intoxication. Cell rounding curves at 8 hours post CDT intoxication (same experiments as in Figure 4, data are plotted at the last timepoint).

Supplementary Figure 7

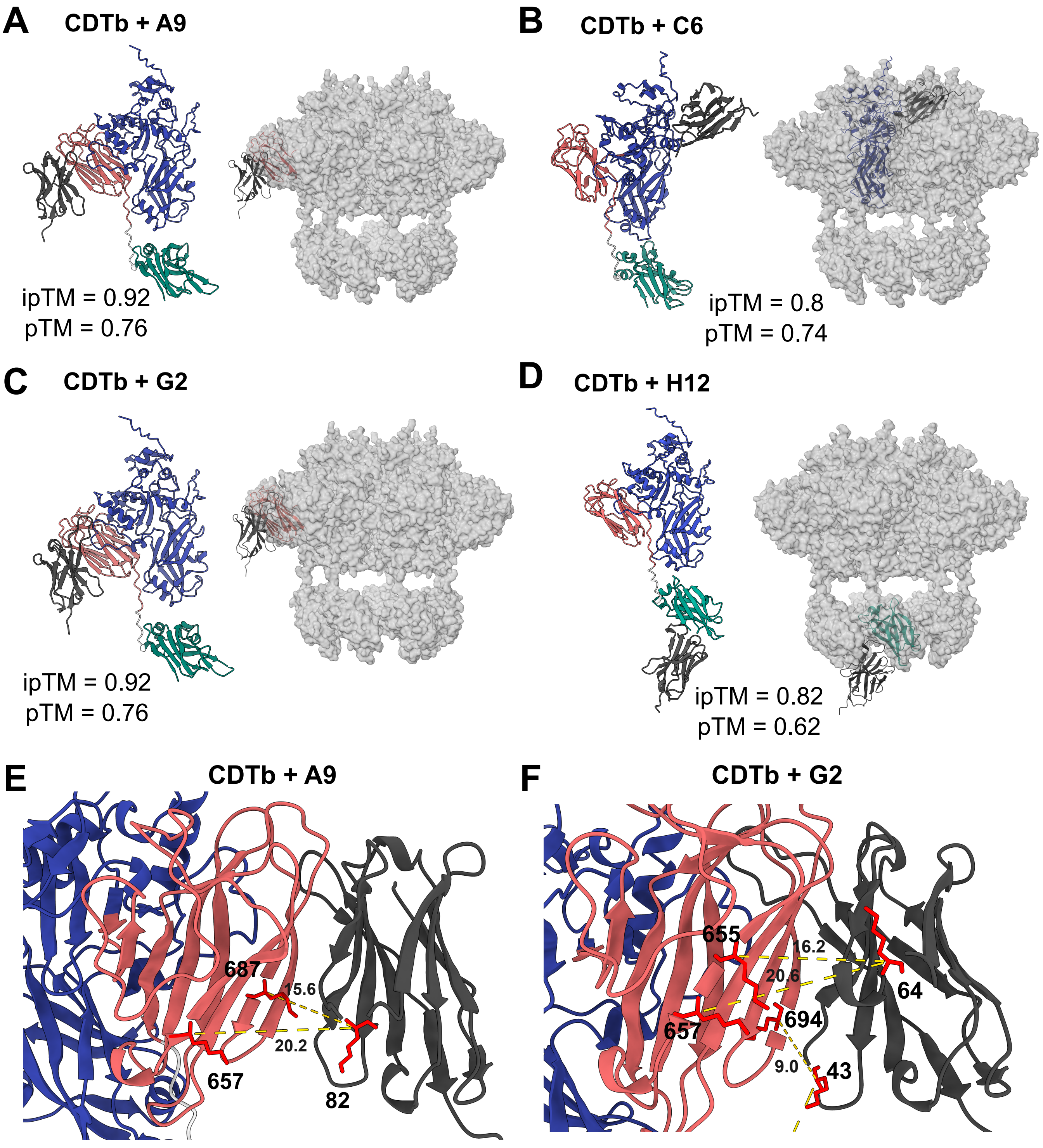

Figure S7. Epitope mapping helps visualize and predict mechanisms of neutralization. AlphaFold3 models of (**A**) CDTb+ A9, (**B**) CDTb + C6, (**C**) CDTb + G2, and (**D**) CDTb+ H12 complexes. CDTb is colored based on domain specificity that was assigned previously (blue -

D1-D3, red - D3’, and green - D4). Nbs are shown in grey. Predicted template modeling (pTM) and interface predicted template modeling (ipTM) scores are shown next to each complex. Complete AlphaFold3 models with pLDDT and predicted aligned error (PAE) scores are shown in the Supporting Material. Nanobody interactions with appropriate domains were fitted into a CDTb heptamer to visualize whether these interfaces interfere with the adjacent protomers within CDTb heptamer. Distance measurements of (**E**) CDTb+A9 and (**F**) CDTb+G2 crosslinks mapped onto the CDTb+A9 and CDTb+G2 AlphaFold3 models shown in (**A**) and (**C**). Distances (in Å) between the two crosslinked α-Carbons of Lysine residues (in red) are shown in yellow dotted lines and are summarized in Supplementary Tables 3 and 4.

Supplementary Figure 8

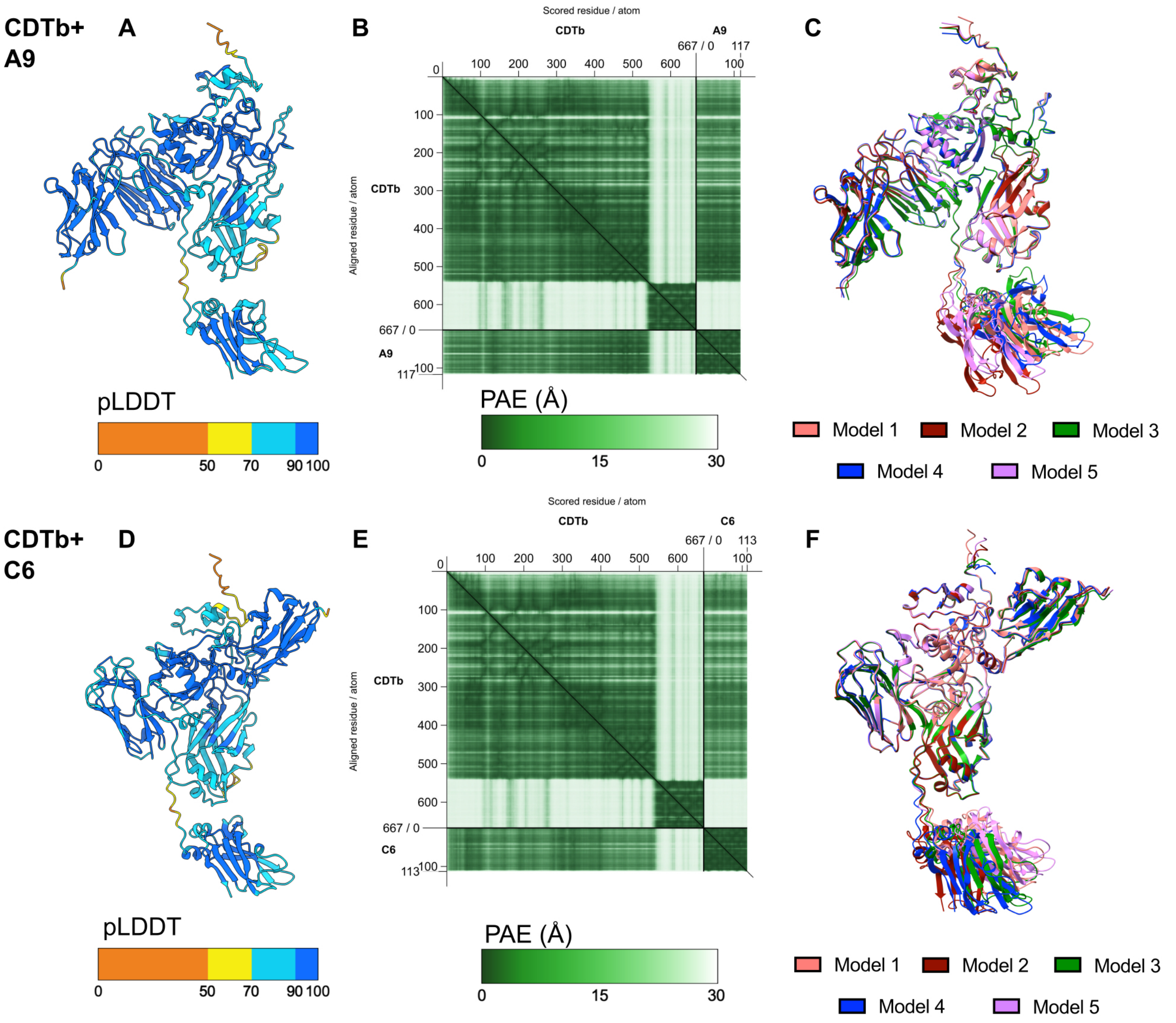

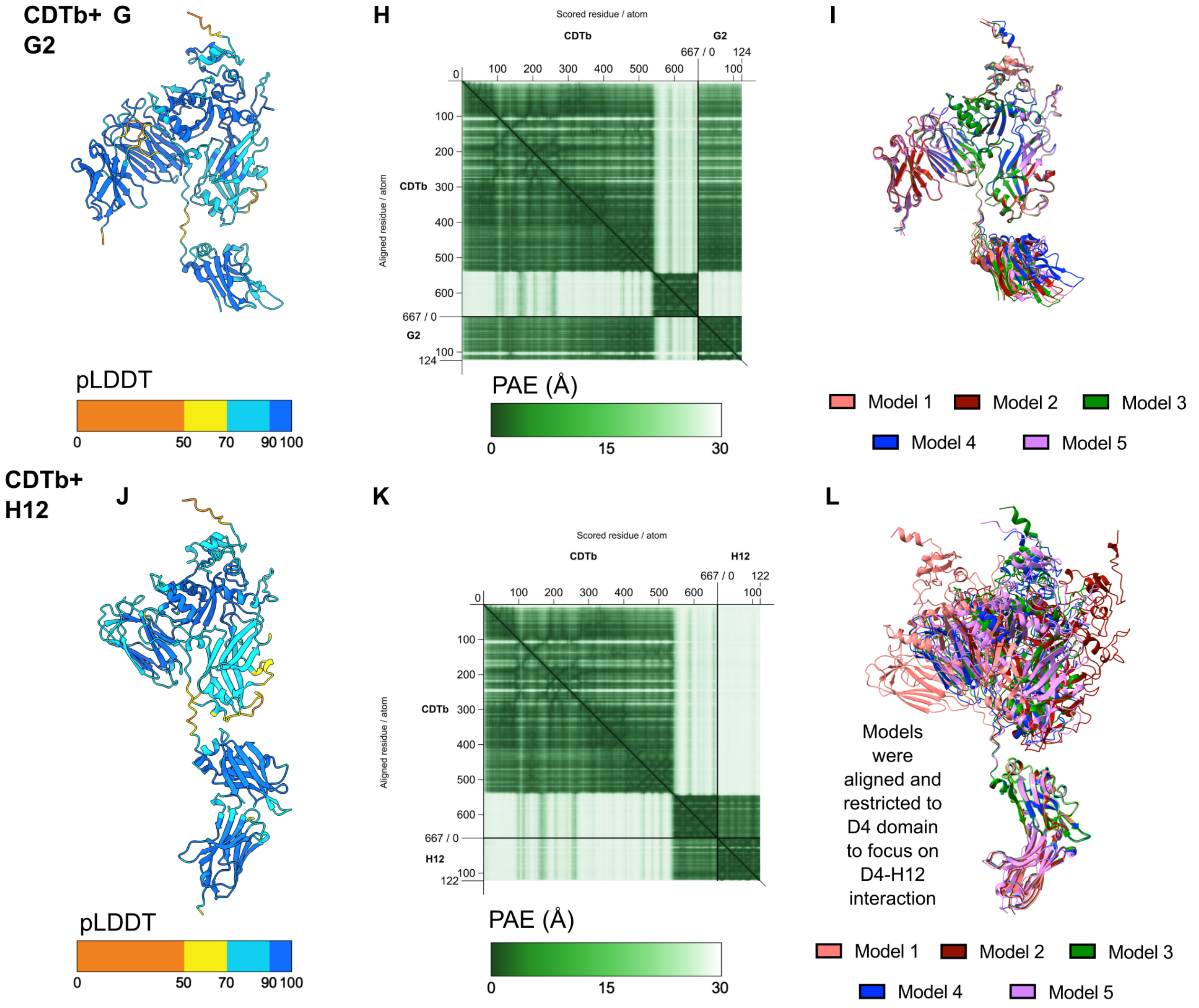

Figure S8. Confidence metrics of AlphaFold3 models. pLDDT (predicted local distance difference test) values, predicted aligned error (PAE) plots, and an overlay of 5 AlphaFold3 models showcasing uniformity of the prediction are shown respectively for CDTb+A9 (**A – C**), CDTb+C6 (**D – F**), CDTb+G2 (**G – I**), and CDTb+H12 (**J – L**). According to AlphaFold3, pLDDT > 90 = very high, 90 > pLDDT > 70 = confident, 70 > pLDDT > 50 = low, pLDDT < 50 = very low confidence predictions. PAE matrix was visualized and extracted from PAE Viewer. Model overlay shows high uniformity of the prediction and differs mainly in the angle placement of D4. For CDTb+H12, models were restricted to D4 domain to focus on the uniformity of D4-Nb interaction.

Supplementary Figure 9

A CDTb (K657)-A9(K82)

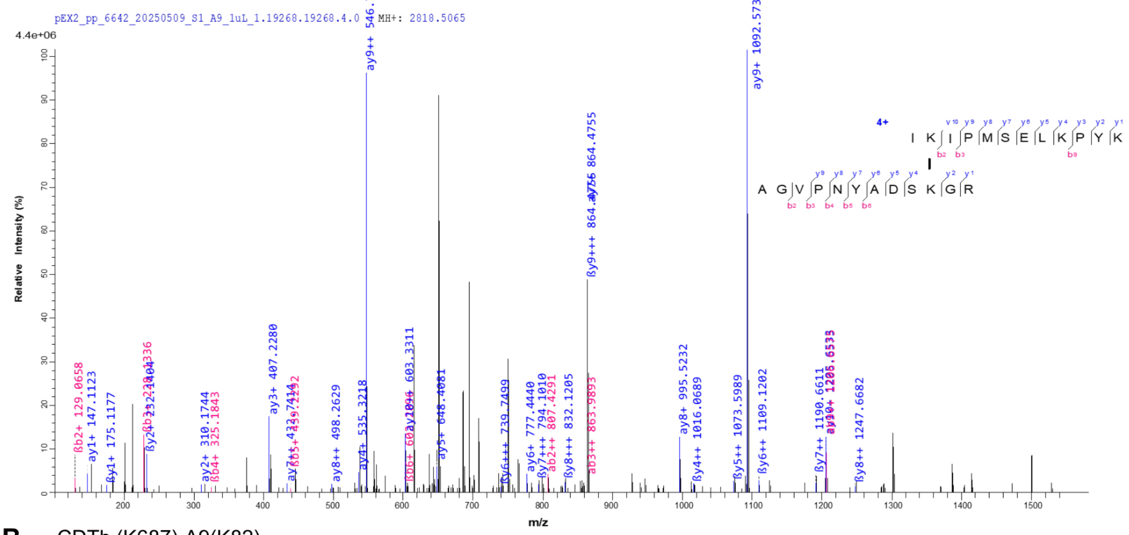

B CDTb (K687)-A9(K82)

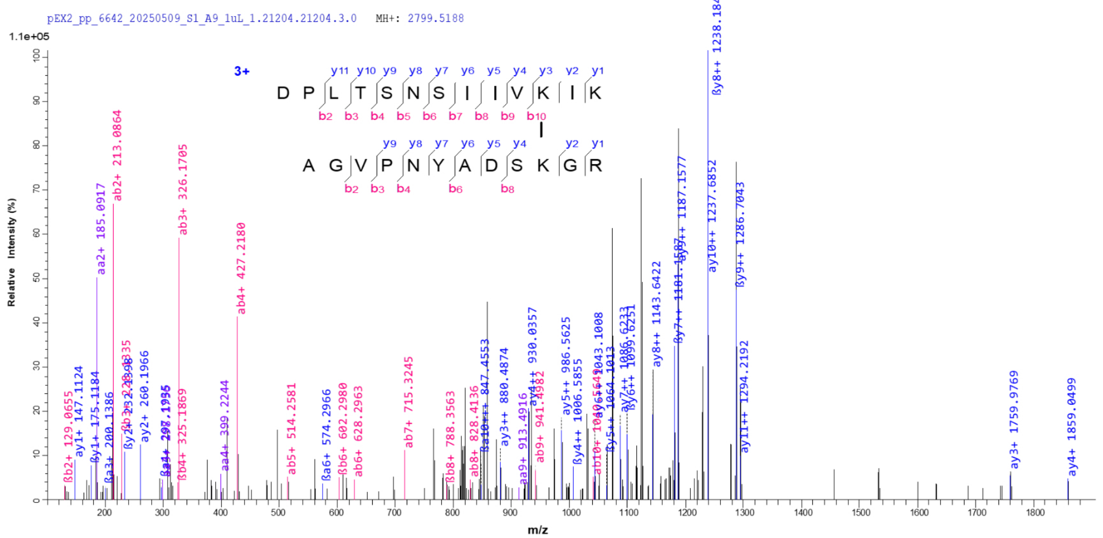

C G2(K83)-CDTb (K655)

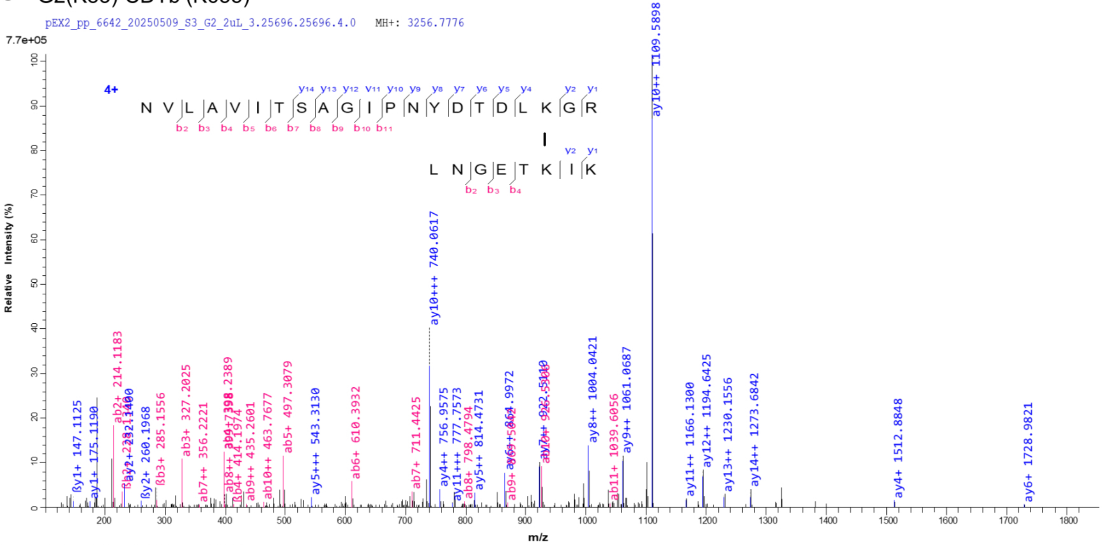

D G2(K83)-CDTb(K657)

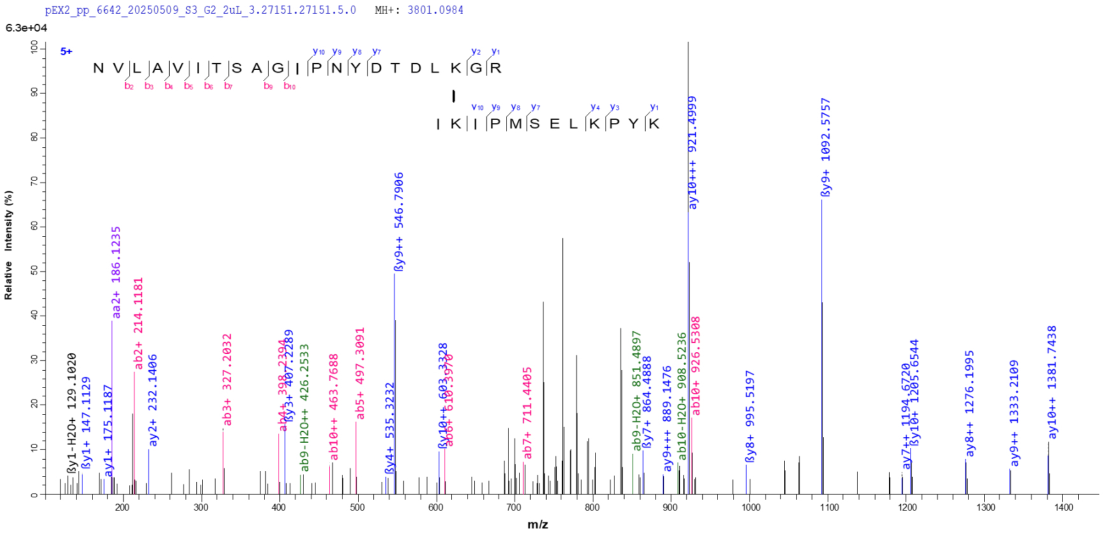

E CDTb(K694)-G2(K62)

pEX2_pp_6642_20250509_S3_G2_2uL_3. 15037. 15037. 4. 0 MH+: 2929. 5037

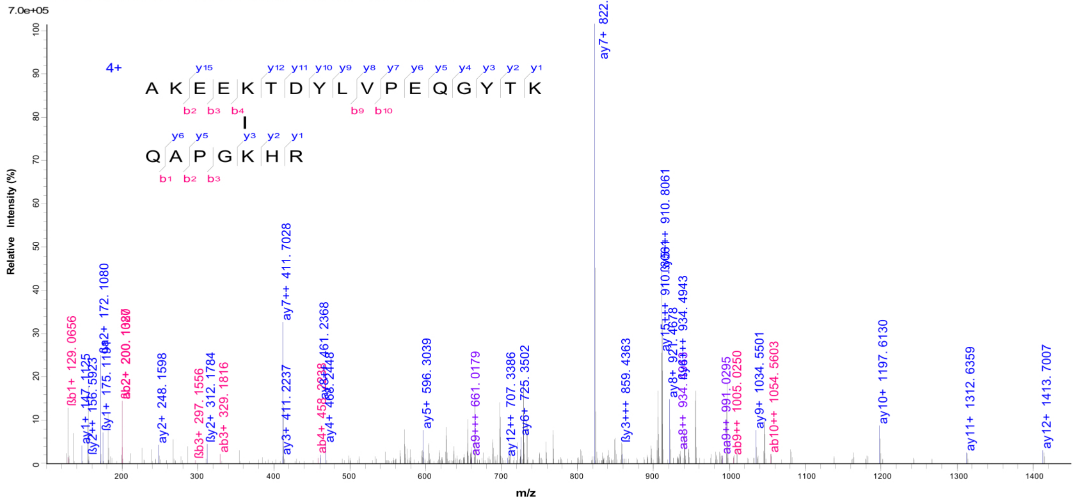

F CDTb(K694)-G2(K62)

pEX2_pp_6642_20250509_S3_G2_2uL_3. 16215. 16215. 3. 0 MH+: 2730. 3710

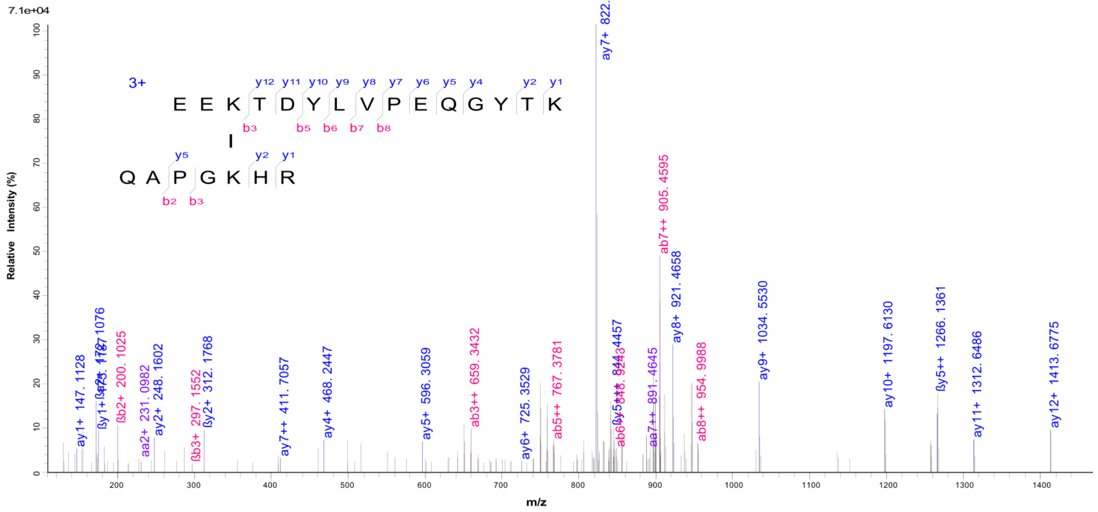

G G2(K62)-CDTb(K821)

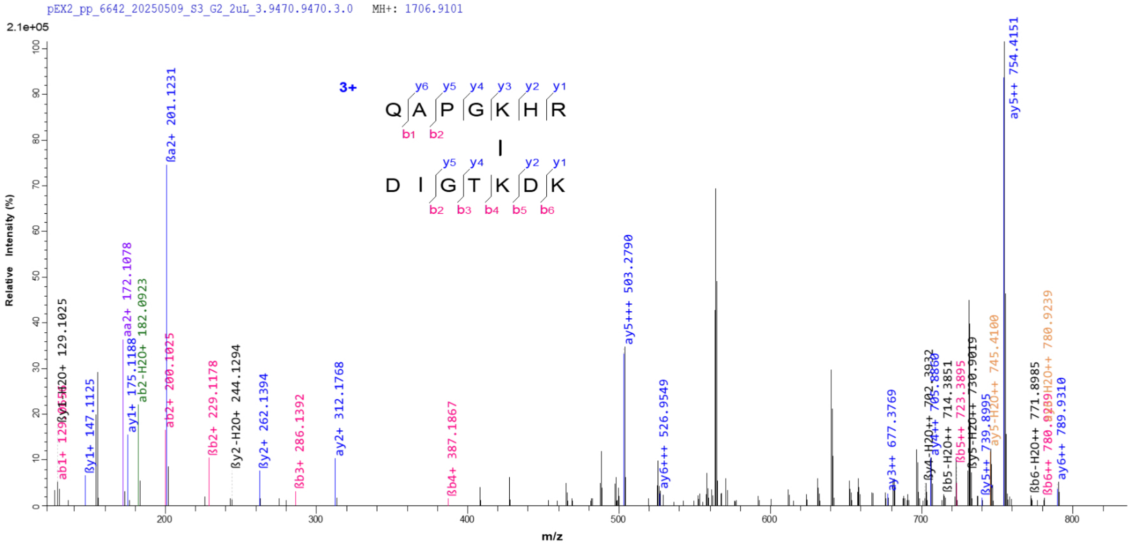

H CDTb + G2

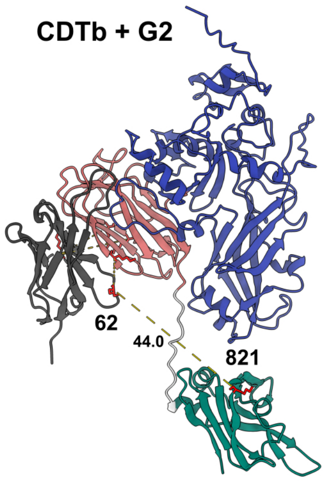

Figure S9. Tandem mass spectra of crosslinked peptides between CDTb and nanobodies. **(A)** Quadruply charged crosslinked peptide $[M+4H]^{4+}$ between CDTb (K657) - A9(K82). **(B)** Triply charged crosslinked peptide $[M+3H]^{3+}$ between CDTb (K687) - A9(K82). (**C**) Quadruply charged crosslinked peptide $[M+4H]^{4+}$ between G2(K83) - CDTb (K655). (**D**) Quintuply charged crosslinked peptide $[M+5H]^{5+}$ between G2(K83) - CDTb(K657). (**E**) Quadruply charged crosslinked peptide $[M+4H]^{4+}$ between CDTb(K694) - G2(K62). (**F**) Triply charged crosslinked peptide $[M+3H]^{3+}$ between CDTb(K694) - G2(K62). (**G**) Triply charged crosslinked peptide $[M+3H]^{3+}$ between G2(K62) - CDTb(K821). The amino acid sequences are provided above each spectrum, and the first and last amino acid residues of the peptides are summarized and labeled in Supplementary Tables 3 and 4. Crosslinked peptides are marked with a line. (**H**) G2(K62) - CDTb(K821) crosslink mapped onto the CDTb+G2 AlphaFold3 model.

Supplementary Figure 10

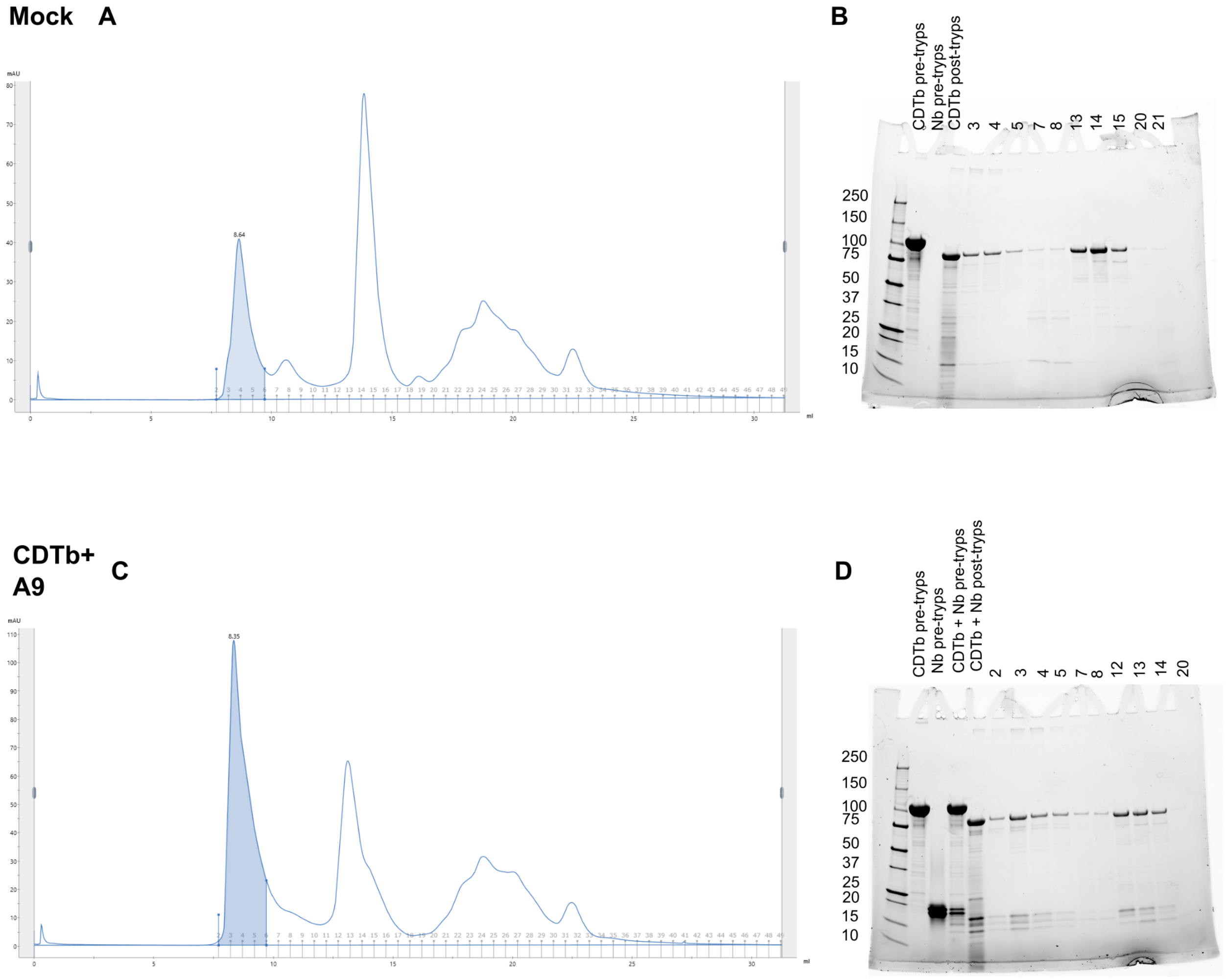

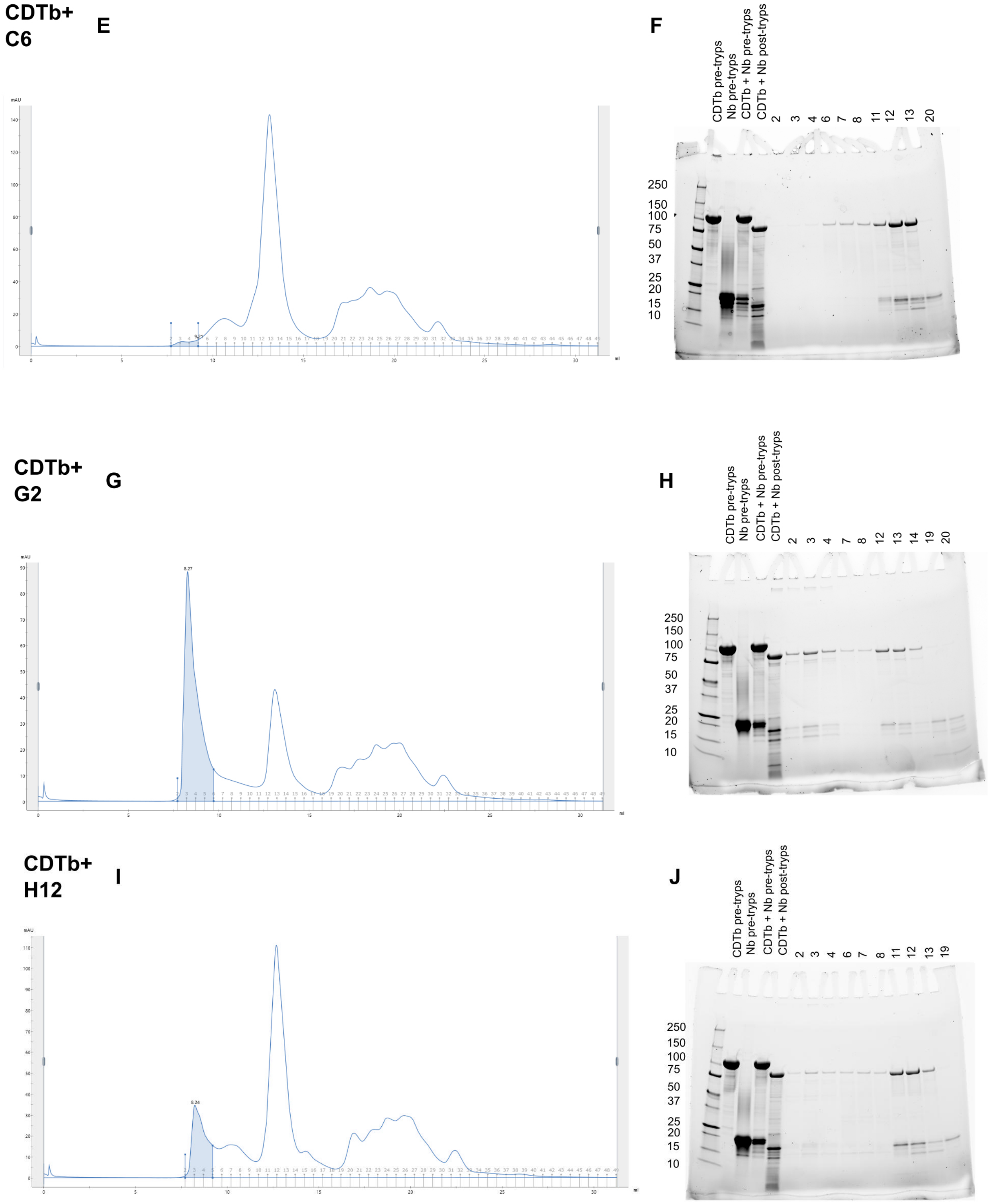

Figure S10. Representative chromatographs and accompanying SDS-PAGE gels for oligomerization assay. Data for (**A - B**) mock (CDTb alone), (**C - D**) CDTb + A9, (**E - F**) CDTb + C6, (**G - H**) CDTb + G2, and (**I - J**) CDTb + H12 reactions. Area highlighted in blue corresponds to double heptamer peak that was used for double heptamer yield quantification.

SDS-PAGE gels were labeled according to the fractions on the chromatographs. Equal volume of sample was loaded across all gels.

Supplementary Figure 11

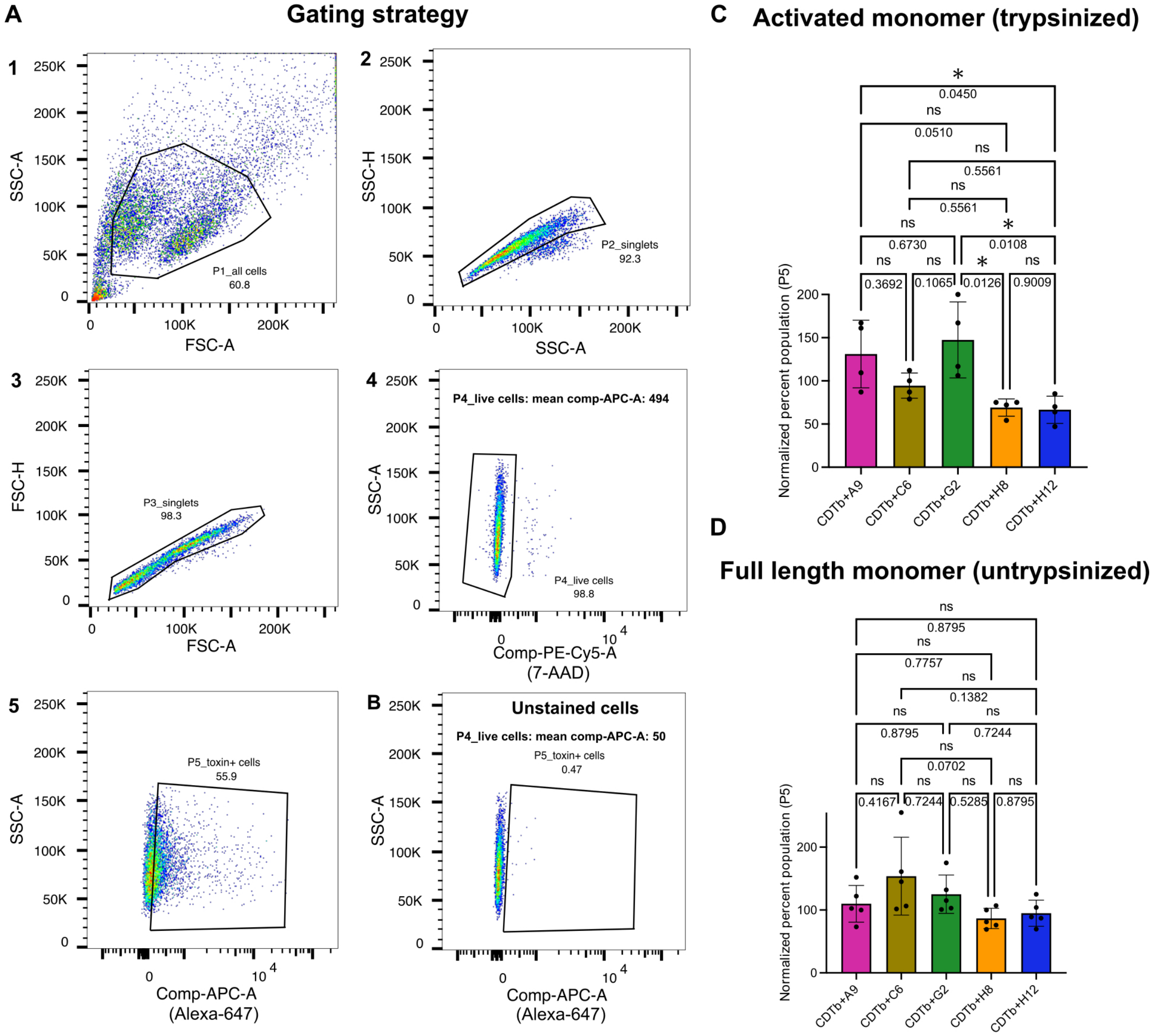

Figure S11. Flow cytometry gating strategy. (**A**) Gating strategy is exemplified on 7AAD⁺ CDTb-Alexa647⁺ cells: 1) – cells were selected according to side (SSC) vs forward (FSC) scatter areas (two populations were consistently observed between all independent experiments and gate was placed consistently across both populations in all experiments), 2) – singlets were selected according to SSC height (H) vs SSC-A gate, 3) - singlets were further selected according to FSC-H vs FSC-A gate, 4) - live cells were selected as a double negative population according to SSC-A vs PE-Cy5-A gate (PE-Cy5-A$^+$ cells are 7-AAD$^+$ meaning that the cell membrane integrity was lost allowing 7-AAD to penetrate and stain dead cells, 5) – CDTb$^+$ cells were selected according to SSC-A vs APC-A gate (APC$^+$ corresponds to Alexa-647$^+$). Mean fluorescent intensity (MFI) of APC$^+$ cells is taken from the live cell gate (P4 from step 4). (**B**) APC$^+$ gate was set relative to unstained cells. Percent population frequencies (APC$^+$ P5 cells) of Alexa647$^+$ cells treated with (**C**) trypsinized CDTb or (**D**) full length (pro) CDTb correspond to MFI data in Figure 5. Bars represent mean ± SD of the group; dots represent an individual independent biological experiment. Each biological experiment consisted of one technical replicate. One-way ANOVA with Holm-Šídák's multiple comparisons test was used to calculate statistical significance. For trypsinized CDTb, the experiment was independently performed 4

times (n = 4). For full length (pro) CDTb, the experiment was independently performed 5 times (n = 5).

Supplementary Figure 12

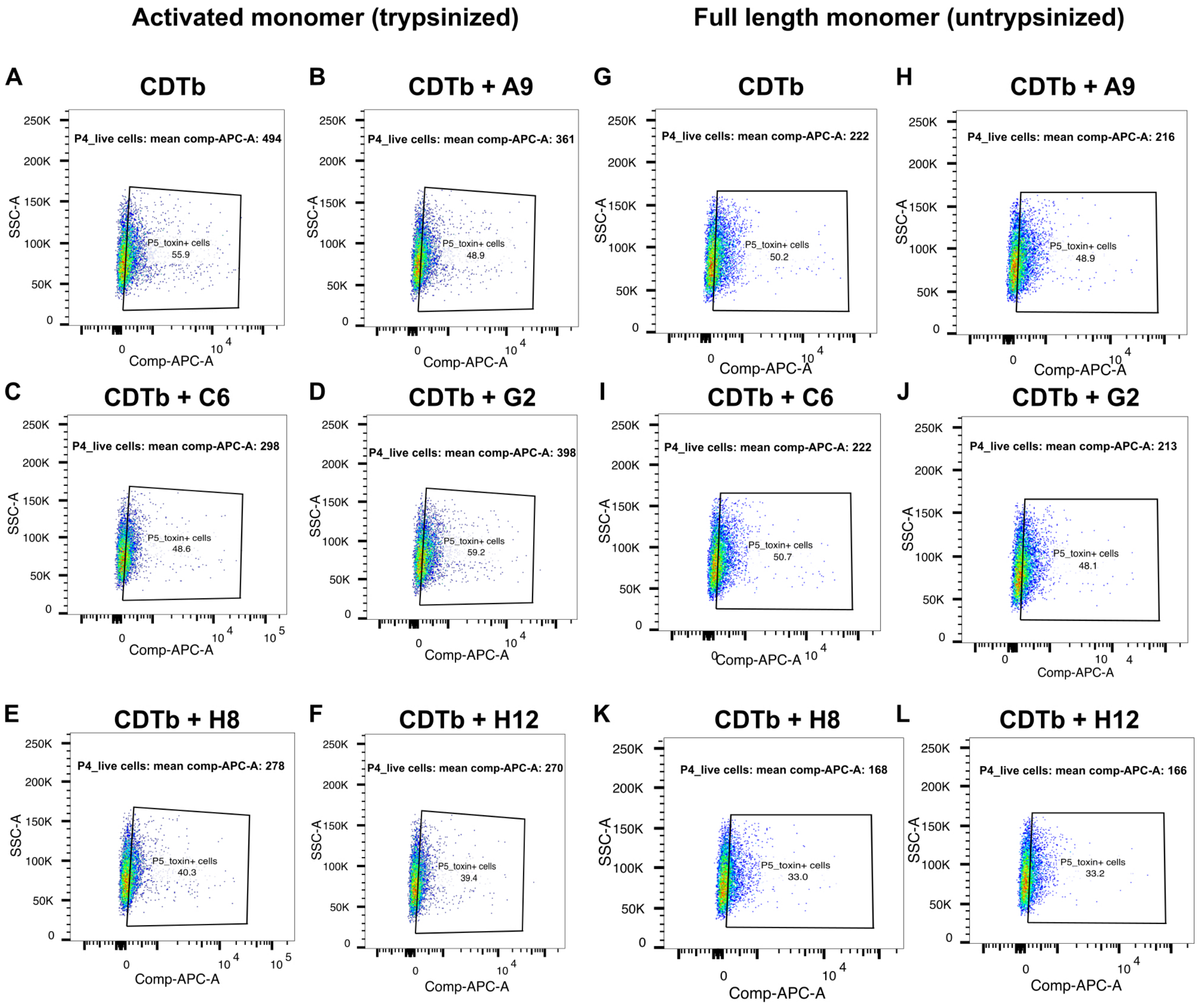

Figure S12. Representative flow cytometry plots. Representative flow cytometry plots of CDTb-Alexa647^{+} (SSC-A vs APC-A gate) cells treated with (**A** and **G**) CDTb only, (**B** and **H**) CDTb + A9, (**C** and **I**) CDTb + C6, (**D** and **J**) CDTb + G2, (**E** and **K**) CDTb + H8, and (**F** and **L**) CDTb + H12. In **A – F**, activated trypsinized CDTb monomer was used. In **G – L**, activated trypsinized CDTb monomer was used.

Supplementary Table 1. Sequences of 63 nanobody clones.

Nb	Sequence
C1A3	QVQLQESGGGLVQAGDSLRLSCAASGRSFSTYAMGWFREAPGKEREFVATFRWFSGITYYAESVKGRFTISRDNAKNTVYLQMNSLKPE DTAVYYCAATRGTTSGYFIPENEGEYDYWGQGTQVTGS
C1A4	QVQLQESGGGLVQAGDSLRLSCAFSGDTFSSYTMGWFRQAPGKEREFVAAFSGIGTGTYYADYVKGRFTISRDNAKNTLYLQMNNLKPE DTAVYYCAATTGGTYYIFELETEYDYWGQGTQVTGS
C1A5	QVQLQESGGGLVQPGGSLRLSCAASGFTFVDYAMGWIRQATGKNNEGVSCVSGSGRMTDYADSVKGRFTISRDNAKNMVYLQMNNLK PEDTGVYYCVASWDDGYGXSCITPEYRGQGTQVTGS
C1A6	QVQLQESGGGLVQPGGSLRLSCVAAGTDFKHNLMGWYRQAPGKQRELVASVLGSGGGGLITYGDPVKGRFTISRDSAQNSVDLQMNNL HPEDTAIYYCHAKKMGTDYWGPGTQVTGS
C1A7	QVQLQESGGGLVQAGGSLRLSCANSGRTLNSFAMGWFRQAAGKEREFVAAISRIAGTSYADSVKGRFTISKDYAKNTLYLQMTSLKPEDT AVYYCAATAPSGYVILEMDTEYDYWGQGTQVTGS
C1A8	QVQLQESGGGLVQPGGSLRLSCTTSGFTFSTYNMKWYRQAQGKERELVATISNANNLISYAPSVRGRFTISRDNAKNTLYLQMNSLKPED TAEYYCNAQNFLSNYWGQGTQVTGS
C1A9	QVQLQESGGGLVQPGGSLRLSCTSSTNMLEFTAVAWWRQAPGKQRDLLAVITRAGVPNYADSKGRFAISRDNAKNTVDLLINTLEPDDTA VYTCNARTSGNINYWGQGTQVTGS
C1A11	QVQLQESGGGLVQAGDSLRLSCAASGRTFSTYAMGWFRETPGKEREFTFRWFSGRTYYADSVKGRFTISRDNAKNTVYLQMNSLKPED TAMYYCAATAGTTSGYFIPENDGEYDYWGQGTQVTGS
C1A12	QVQLQESGGGSVEIGGSVNLSCAASGGTFRRYTMAWFRQVPGKSREFVAARQRDGLILYEPSVKGRFAISRDNAENTLTLQMNNLRVED TAIYYCAARDIMAFSRDPQNYDYWGQGTQVTGS
C1B1	QVQLQESGGGLVQAGDSLTLSCAASGRTFSTYAMGWFREAPGKEREFVATVRWFLGNTYYADSVKGRFSISKDNAKNTVYLQMNSLKPE DTAVYYCAATKGTTSGYFIPENDGEYDYWGQGTQVTGS
C1B2	QVQLQESGGGLVQPGGSLRLSCADSGTTFIYYAMGWFRQAPGKEREVVACINGNGDNPYYADSVRGRFTISRDDAKNTVYLQMNGLKP EDAAVYYXAAANDFDGRCYXXSLWYXDWXQGTXVTGS
C1B3	QVQLQESGGGSVQAGGSLKLSCAASAGTFSNYALGWFRQAPGKEREFVAHISWIGGRTNYADSVKGRFTISRDNAKNTLYLQMNGLEIE DTAVYYCAACRGGAYYVFSHAAEYDYWGQGTQVTGS
C1B4	QVQLQESGGGLVQPGGSLRLSCTDAGTTAIYYAIGWFRRAPGKEREGIACINMNGDGTNYADSVKGRFTISRDDAKNTVYLQMNSLKSED AAVYYCAAANDFDGRCNLRSPWYSDWGQGTQVTGS
C1B5	QVQLQESGGGLVQPGGSLTLSCAASGSIGSGSVMSWYRQAPGKERELVAQESGGRLRSYAKSVMGRFTISRDNSNNTVTLQMNNLKTD DTAIYYCHLVQFSGRYWGHGTQVTGS
C1B6	QVQLQESGGGLVQSGGSLRLSCAASDSTFSNYALGWFRQAPGKEREFVAHIRWIGGSTEYANSVKGRFTISRDNAKSTLYLQMNSLKPE DTAVYYCAAVRGGSYYIASSEAEYDYWGQGTQVTGS
C1B7	QVQLQESGGGLVQPGGSLRLSCTSSTNMLEFTAVAWWRQAPGKQRDLLAVITRAGVPNYADSKGRFAISRDNAKNTVDLLINTLEPDDTA VYTCNARTSGNINYWGQGTQVTGS
C1B9	QVQLQESGGGLVQPGGSLRLSCVASGFTFSRYDMSWHRQAPGKERELVASTRTMTGTGVNYADSVKGRFTMSIDGAKNTVYLQMNSLK PEDTAVYYCHAERKYAYYDPEYDYWGQGTQVTGS
C1B11	QVQLQESGGGLVQAGDSLRLSCAASERTFRTYAMGWFREAPGKEREFVAALRWFLGNTYYADSVKGRFTISKDDAKNTLYLLMNSLQPD DTAVYYCAATHGTTSGYFIPDNEGEYDYWGQGTQVTGS
C1B12	QVQLQESGGGLVQAGDSLRLSCAASGRTFSTYAMGWFREAPGKEREFVATFRWFSGRTYYADSVKGRFTISRDSAKNTVYLQMNSLKP EDMAMYYCAATAGTTSGYFIPENDGEYDYWGQGTQVTGS
C1C1	QVQLQESGGGLVQPGGSLRLSCVASGGIFSIYAMSWYRQAPGKQRELVATIATYGTADYEDAVKGRFTISRDNAKNTVYLQMNSLKPEDT AVYYCYADPEMGVAGMKDYWGQGTQVTGS

C1C3	QVQLQESGGGLVQAGGSLRLSCAASGRTFSSYAAGWFRQAPGNEREFVSAINKLGTSTYYEDSVKGRFTISRDNAKNTLYLEMNSLKPEDTAVYYCAATADPSWYTMPGNEGEYDYWGQGTQVTGS
C1C4	QVQLQESGGGLVQPGGSLRLSCAASGYTINSLTVAWFRQAPGKEREFIAASGAPDNLTGYQNSLKGRFTISRDNAKNTLYLQMTSLKPEDTAVYYCAARPGPYSSQTRDYPYWGQGTQVTGS
C1C5	QVQLQESGGGLVQAGDSLRLSCAFSGDTFSSYTMGWFRQAPGKEREFVAAFSAIGTGTYYADYVKGRFTISRDNAKNTLYLQMNNLKPEDTAVYYCTATTGGTYYIFELETEYDYWGQGTQVTGS
C1C6	QVQLQESGGGLVEPGGSLRLSCVASGIVVASSTMSWYRQAPGKQRELVAQIFSGGNTNYRDSVRGRFTISKDNAKNTAYLQMDNLEPDDTAVYYCYARGFWGQGTQVTGS
C1C10	QVQLQESGGGLVQSGGSLRLSCAASGSPITTLTVAWFRQAPGKEREFIAASGSPDNLTGYTPSLKGRFTISRDNAKNTLYLQMTSLNPEDTAVYYCAARPGPYSSQTRHYPYWGQGTQVTGS
C1C11	QVQLQESGGGTVQAGGSLRISCAASGRTASNYAMGWFRQAPGKEREFVAHIRWLGGSTEYADSVKGRFTISRDNAKNTLYLQMNGLKPEDTAVYYCAAVRGGSYYIHDNEAEYDYWGQGTQVTGS
C1D1	QVQLQESGGGLVQPGRSLRLSCEASDFSLNTFGAGWFRQVPGKEREGVSCISPSNSSTYYADSVKGRFTISRDNAKNTIYLQMNSLKPEDTGVYFCAARPITSAQALCTLLVSDSFTSWGQGTQVTGS
C1D3	QVQLQESGGGLVQPGESLRLSCTASGFTLDRYHIGWFRQAPGKEREGVACISSNLGTTNYADSVKGRFTISRDNAKNTVTLQMNSLTPEDTGVYYCAGQPRANMFAYSFCAPYEYDYWGQGTQVTVS
C1D6	QVQLQESGGGLVQPGGSLRLSCTASGGIFSIYAMNWYRQAPGKQRELVASISTLSTTDYADSVKGRFTISRDNAKNAVYLQMNSLKPEDTAVYYCNADPEMGVAGMREYWGQGTQVTGS
C1D7	QVQLQESGGGLVQAGDSLRLSCANSGRTFSSYGLAWFREAPGKEREFVAALRWGNPYYADSVKGRFTISRDNAKTTLYLQMNSLKPEDTGVYYCAATEGSSGYFIAENEGEYDYWGQGTQVTGS
C1D8	QVQLQESGGGLVQAGDSLRLSCAASEGTFETYGMGWFRQAPGREREFVASVNWLGGNVVYAPSVKGRFTISRDNTKNMLYLQMNSLNPMDTAVYYCAASQSQYQVGFTTRSADFGSWGQGTQVTGS
C1D9	QVQLQESGGGLAQTGNSLRLSCAASGGTIGTLTVAWFRQAPGKEREFVAATGVPDNLTGYTPSLKGRFTISRDNAKNALYLQMTTLKPEDTAVYYCAARPGPYSSRTTDYPYWGQGTQVTGS
C1D10	QVQLQESGGGLVQPGGSLRLSCAVSGLAXDYYAIGWFRQAPGKEREEVSCLNGSDGXTYYGDSVKGRFTISRDNAKNTVYLQLNRLXPEDXGVYYCAAXRGGWGCLTDSFSXAEYDYWGQXTQVTGS
C1D11	QVQLQESGGGLLQPGGSLRLSCVASGFTFSTATIKWYREAPEKERELVALIGNTGGLTSYAPSVKGRFTVSRDNAKSTVWLQMNTLKPEDTAMYFCNAQISGAPYFGENFWGQGTQVTGS
C1D12	QVQLQESGGGLVQPGESLRLSCTASGFTLDRYHIGWFRQAPGKEREGVACISSNLGTTNYADSVKGRFTISRDNAKNTVTLQMNSLTPEDTGVYYCAGQPRANMFAYSFCAPYEYDYWGQGTQVTVS
C1E4	QVQLQESGGGLVQPGGSLRLSCVVSGSTLDHYTIGWFRQAPGKEREGVAWISMNYGRTNYADSAKGRFTISRNNAENTVYLQMTSLEPMDTAVYYCAAEDSTYGSAWPNDWXMDYWGPGTQVTGS
C1E5	QVQLQESGGGLAQPGGSLRLSCEVSGFDFSSSDMSWHRQTPGNERELVAAIASRSGLISYKDSVKGRFTVSRDNAKNTVYLQMSSLKPEDTGTYYCNVGFRVSSLASWGQGTQVTVS
C1E6	QVQLQESGGGLVQAGDSLRLSCANSGRTFSSYGLAWFREAPGKEREFVAALRWGNPYYADSVKGRFTISRDNAKTTLYLQMNSLKPEDTGVYYCAATEGSSGYFIAENEGEYDYWGQGTQVTGS
C1E7	QVQLQESGGGLVQAGDSLRLSCANSGRTFSSYGLAWFREAPGKEREFVAALRWGNPYYADSVKGRFTISRDNAKTTLYLQMNSLKPEDTGVYYCAATEGSSGYFIAENEGEYDYWGQGTQVTGS
C1E9	QVQLQESGGGLVLAGDSLRLSCAASGRTFSTYAMGWFREAPGKEREFVATIRWFLGNTYYADSVKGRFTISKDNAKNTIYLQMNSLKPEDTAMYYCAATAGTTSGYFIPENEGEYDYWGQGTQVTGS
C1E10	QVQLQESGGGLVQTGDSLTLSCAASGRTFSTYAMGWFREAPGKEREFVATVRWFLGNTYYADSVKGRFSISKDNAKNTVYLQMNSLKPEDTAVYYCAATKGTTSGYFIPENDGEYDYWGQGTQVTGS
C1E11	QVQLQESGGAMVQPGGSLRLSCAASGSGSNSSAMAWYRQAPGKQRELIAVITRANVPNYVPSMKDRFTISRDNAKNTAYLQIDSLKAEDTAVYYCNARTTTGVNYWGQGTQVTGS

C1F5	QVQLQESGGGLVEPGGSLRLSCVASGIVVASSTMSWYRQAPGKQRELVAQIFSGGNTNYRDSVRGRFTISKDNAKNTAYLQMDNLEPDDTAVYYCYARGFWGQGTQVTGS
C1F7	QVQLQESGGGLVQTGDSLRLSCEVSGATFGDYRLSTAWFRQAPGKDREFVAIIMRLGDTYYSDSMKGRFTISRDDTGNTLYLQMNGLNPEDTAVYFCAAGEQYYCSPDRCKNTNDYDYWGQGTPVTGS
C1F8	QVQLQESGGGLVQAGGSLRLSCANSGRTLNSFAMGWFRQAAGKEREFVAAISRIAGTSYADSVKGRFTISKDYAKNTLYLQMTSLKPEDTAVYYCAATAPSGYVILEMDTEYDYWGQGTQVTGS
C1F9	QVQLQESGGGLVQPGGSLRLSCTASGSTSDLYTTGWFRQAPGKEREGVSCISFSSGSTDYADSVKGRFTISRDDAKNTVYLQMNNLKPEDTAVYYCGLIAWLEGDGSSCLKRAWEYDYWGQGTQVTGS
C1F10	QVQLQESGGGSVQPGGSLRLSCAPTGSSLDYYAIGWFRQAPGKEREVVATITNTGGLTSYADSVKGRFTISRDNAKNTVYLQMNSLKFEDTAVYYCELRNVGNTYWGQGTQVTGS
C1F11	QVQLQESGGGLVQPGGSLRLSCAASADISVYTTLGWYRQAPGKERELVAQTTSRGDTDYADSVKGRFTISRDNAKNMLYLXMDSLKPEDTAVYYCNAKYTVNWYENDYWGQGTQVTGS
C1G1	QVQLQESGGGLVQPGGSLTLSCAASGSIGSGSVMSWYRQAPGKERELVAQESGGRLRSYAKSVMGRFTISRDNSNNTVTLQMNNLKTDDTAIYYCHLVQFSGRYWGHGTQVTGS
C1G2	QVQLQESGGGLVQPGGSLTLSCKASGIVFSTHTLAWYRQAPGKHRNVLAVITSAGIPNYDTDLKGRFTISRDNAKNTVYLQMNSLEPEDTAVYYCNARGPHPTVTGTTANYWGQGTQVTGS
C1G4	QVQLQESGGGLVQAGDSLTLSCAASGRTFSTYAMGWFREAPGKEREFVATVRWFLGNTYYADSVKGRFSISKDNAKNTVYLQMNSLKPEDTAVYYCAATKGTTSGYFIPENDGEYDYWGQGTQVTGS
C1G5	QVQLQESGGGLVQAGDSLRLSCAASEHTFSNYALAWFRQVPGQEREFVAHISWIGGKTNYADSVKGRFTISRDNAKNTLYLQMNSLKPEDTAVYYCAACRGGSYYIHSSEAEYDYWGQGTQVTGS
C1G6	QVQLQESGGGLVQPGGSLRLSCASSGFAFSKYSMTWVHQAPGKGLEWVSQITGSADNIYYADSVKGRFIISRDNAKNTLYLQMNSLKPEDTGVYYCAIDIRWDNAYWGQGTQVTGS
C1G8	QVQLQESGGGLVQAGDSLRLSCAASGRTFSGYAMGWFRQAPGKEREVVAAISWIAGSTHYADFAKGRFTISRDKAKNTLFLQMNSLKPEDTAVYYCAAAGKENPYPLIWEEYEYDYWGQGTQVTGS
C1G10	QVQLQESGGGLVQAGGSLXLSCAASGGTFSNYALGWFRQAPGKERELVAHISWIGGPTNYADSVKGRFTISRDNAKNTLYLLMNSLEIEDTAVYYCAACHGGAKYLFSHSAEYDFWGQGTHVTGS
C1H2	QVQLQESGGGLVQPGGSLRLSCAASGFSLDYHAIGWFRQAPGKEREGVSCITSTGVTNYADSAKGRFTISRDNLRNTMYLQMNSLKPEDTAVYYCAAAPPGQLYRLCEPYEYDYWGQGTQVTGS
C1H6	QVQLQESGGGLVQPGGSLTLSCAASGSIGSGSVMSWYRQAPGKERELVAQESGGRLRSYAKSVMGRFTISRDNSNNTVTLQMNNLKTDDTAIYYCHLVQFSGRYWGHGTQVTGS
C1H7	QVQLQESGGGLVQPGRSLRLSCEASDFSLNTFGAGWFRQVPGKEREGVSCISPSNSSTYYADSVKGRFTISRDNAKNTIYLQMNSLKPEDTGVYFCAARPITSAQALCTLLVSDSFTSWGQGTQVTGS
C1H8	QVQLQESGGGLVQPGRSLRLSCEASDFSLNTFGAGWFRQVPGKEREGVSCISPSNSSTYYADSVKGRFTISRDNAKNTIYLQMNSLKPEDTGVYFCAARPITSAQALCTLLVSDSFTSWGQGTQVTGS
C1H9	QVQLQESGGGSVQAGGSLKLSCAASAGTFSNYALGWFRQAPGKEREFVAHISWIGGRTNYADSVKGRFTISRDNAKNTAYLQMDNLEPDDTAVYYCYARGFWGQGTQVTGS
C1H10	QVQLQESGGGLVQAGDSLRLSCAASGRTYARAWFRESPGKEREFVAAIGIGGITTYYANSVKGRFTISRDNAKNTLYLQMDSLKPEDTAVYYCAATGGSIGYFIAENEAEYDYWGQGTQVTGS
C1H11	QVQLQESGGGLVQPGGSLRLSCAASGFTFGSYVVQWYRQPPGKERELVAQIRVTDNLISYKNFAKGRFTISTDNTKNTVYLQMNSLTPEDTAVYYCRYQDNWGQGTQVTGS
C1H12	QVQLQESGGGLVQPGGSLRLSCAASGDIFSFYTMGWYRQAPGKQRELVATITSPGTTNYADSVKGRFTISRDNAKNMVYLQMTSLKPEDTAVYYCNADRILTTDPQFKYWGQGTQVTGS

Supplementary Table 2. Sequences of 5 nanobody clones further characterized in the study.

Lab code	Nb	Sequence
**pNb269**	**C1A9**	MGWSCIILFLVATATGVHSQVQLQESGGGLVQPGGSLRLSCTSSTNMLEFTAVAWWRQAPGKQRDLLAVITRAGVPNYADSKGRFAISRDNAKNTVDLLINTLEPDDTAVYTCNARTSGNINYWGQGTQVTVSSGSYPYDVPDYAGSGLNDIFEAQKIEWHEGSLEHHHHHH
**pNb271**	**C1C6**	MGWSCIILFLVATATGVHSQVQLQESGGGLVEPGGSLRLSCVASGIVVASSTMSWYRQAPGKQRELVAQIFSGGNTNYRDSVRGRFTISKDNAKNTAYLQMDNLEPDDTAVYYCYARGFWGQGTQVTVSSGSYPYDVPDYAGSGLNDIFEAQKIEWHEGSLEHHHHHH
**pNb273**	**C1G2**	MGWSCIILFLVATATGVHSQVQLQESGGGLVQPGGSLTLSCKASGIVFSTHTLAWYRQAPGKHRNVLAVITSAGIPNYDTDLKGRFTISRDNAKNTVYLQMNSLEPEDTAVYYCNARGPHPTVTGTTANYWGQGTQVTVSSGSYPYDVPDYAGSGLNDIFEAQKIEWHEGSLEHHHHHH
**pNb274**	**C1H8**	MGWSCIILFLVATATGVHSQVQLQESGGGLVQPGRSLRLSCEASDFSLNTFGAGWFRQVPGKEREGVSCISPSNSSTYYADSVKGRFTISRDNAKNTIYLQMNSLKPEDTGVYFCAARPITSAQALCTLLVSDSFTSWGQGTQVTVSSGSYPYDVPDYAGSGLNDIFEAQKIEWHEGSLEHHHHHH
**pNb275**	**C1H12**	MGWSCIILFLVATATGVHSQVQLQESGGGLVQPGGSLRLSCAASGDIFSFYTMGWYRQAPGKQRELVATITSPGTTNYADSVKGRFTISRDNAKNMVYLQMTSLKPEDTAVYYCNADRILTTDPQFKYWGQGTQVTVSSGSYPYDVPDYAGSGLNDIFEAQKIEWHEGSLEHHHHHH
	**Secretion signal**	MGWSCIILFLVATATGVHS
	**HA tag**	YPYDVPDYA
	**Avi tag**	GSGLNDIFEAQKIEWHE
	**His tag**	HHHHHH

Supplementary Table 3. Summary of CDTb-A9 crosslinked peptides

CDTb XL peptide (residues)	A9 XL peptide (residues)	Crosslinked residues	Distance (Å)
I**K**IPMSELKPYK (656 – 667)	AGVPNYADS**K**GR (73 – 84)	K(657) - K(82)	20.2
DPLTSNSIIV**K**IK (677 – 689)	AGVPNYADS**K**GR (73 – 84)	K(687) - K(82)	15.6

Supplementary Table 4. Summary of CDTb-G2 crosslinked peptides

CDTb XL peptide (residues)	G2 XL peptide (residues)	Crosslinked residues	Distance (Å)
LNGET**K**IK (650 – 657)	NVLAVITSAGIPNYDTDL**K**GR (65 – 85)	K(655) - K(83)	16.2
I**K**IPMSELKPYK (656 – 667)	NVLAVITSAGIPNYDTDL**K**GR (65 – 85)	K(657) - K(83)	20.6
AKEE**K**TDYLVPEQGYTK (690 – 706)	QAPG**K**HR (58 – 64)	K(694) - K(62)	9.0
EE**K**TDYLVPEQGYTK (692 – 706)	QAPG**K**HR (58 – 64)	K(694) - K(62)	9.0
*DIGT**K**DK (817 – 823)	QAPG**K**HR (58 – 64)	K(821) - K(62)	N/A

*This crosslink involves the D4-G2 interaction (Supplementary Figure 9H). We think that the flexibility of D4 in solution most likely placed D4 near the G2 nanobody resulting in a random crosslink within an appropriate distance constraint.
